# Supplementary material for: Discovery of a Streptococcus pneumoniae serotype 33F capsular polysaccharide locus that lacks wcjE and contains a wcyO pseudogene
Source: PLoS One. 2018 Nov 5;13(11):e0206622. doi: 10.1371/journal.pone.0206622 (PMC6218050; doi:10.1371/journal.pone.0206622)
Supplement: S1 Table — (DOCX) [file pone.0206622.s001.docx]

**S1 Table. Quellung serotyping for Statens Serum Institut (SSI) reference strains (33F and 33A) and two representative 33F-1 isolates.**

| **Factor serum** | **33F** | **33A** | **PMP1348** | **PMP1387** |
| --- | --- | --- | --- | --- |
| 33b | + | + | + | + |
| 33e | - | - | - | - |
| 33f | - | - | - | - |
| 6a | - | - | - | - |
| 20b | - | + | - | - |
