# Supplementary material for: Discovery of a Streptococcus pneumoniae serotype 33F capsular polysaccharide locus that lacks wcjE and contains a wcyO pseudogene
Source: PLoS One. 2018 Nov 5;13(11):e0206622. doi: 10.1371/journal.pone.0206622 (PMC6218050; doi:10.1371/journal.pone.0206622)
Supplement: S1 Fig — Latex reagents were prepared using SSI antisera (33b, 33e, 33f, 6a and 20b) as described in the materials and methods. A positive reaction was defined by the presence of clumping and a reduction in background turbidity. (DOCX) [file pone.0206622.s003.docx]

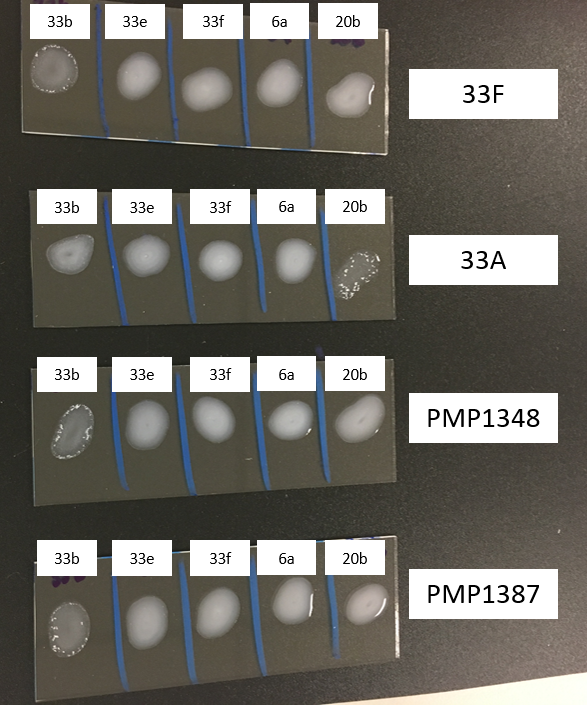


**S1 Fig. Representative latex agglutination reactions of Statens Serum Institut (SSI) 33F and 33A reference strains, and two representative 33F-1 isolates from this study (PMP1348 and PMP1387).** Latex reagents were prepared using SSI antisera (33b, 33e, 33f, 6a and 20b) as described in the materials and methods. A positive reaction was defined by the presence of clumping and a reduction in background turbidity.
