# Supplementary material for: Discovery of a Streptococcus pneumoniae serotype 33F capsular polysaccharide locus that lacks wcjE and contains a wcyO pseudogene
Source: PLoS One. 2018 Nov 5;13(11):e0206622. doi: 10.1371/journal.pone.0206622 (PMC6218050; doi:10.1371/journal.pone.0206622)
Supplement: S2 Fig — These sequences were aligned to a representative sequence from serotype 34 strain 676/74 (Genbank accession no. CR931703), serotype 39 strain 203/40 (Genbank accession no. CR931711) and representative wcyO sequences from serotype 34 and 39 isolates from Fiji using Clustal Omega. Red sequence indicates the 3’end of glf, blue indicates wcyO, and the highlighted regions denote the frameshift mutation site in the 33F-1 sequences. Identical nucleotides are denoted by an asterisk. (DOCX) [file pone.0206622.s004.docx]

Fiji_(34) ------------------------------------------------------------

203/40_(39) ATACTACGACATGCATGTGGTCATTGAACGTGCCTTAGAAGTCGTATTAAGTGAGTTAGG

676/74_(34) ATACTACGACATGCATGTGGTCATTGAACGTGCCTTAGAAGTCGTATTAAGTGAGTTAGG

Fiji_(39) ------------------------------------------------------------

PMP1351_(33F-1) ATACTACGACATGCATGTGGTCATTGAACGTGCCTTAGAAGTCGTATTAAGTGAGTTAGG

PMP1352_(33F-1) ATACTACGACATGCATGTGGTCATTGAACGTGCCTTAGAAGTCGTATTAAGTGAGTTAGG

PMP1379_(33F-1) ATACTACGACATGCATGTGGTCATTGAACGTGCCTTAGAAGTCGTATTAAGTGAGTTAGG

PMP1380_(33F-1) ATACTACGACATGCATGTGGTCATTGAACGTGCCTTAGAAGTCGTATTAAGTGAGTTAGG

PMP1386_(33F-1) ------------------------------------------------------------

PMP1387_(33F-1) ------------------------------------------------------------

Fiji_(34) ----------ATTACATTAAGGTCAAGTTTAATGACCTTATATCTTTTCTATAAAGAAAA

203/40_(39) AAAGTAGTCAATTACATTAAGGTCAAGTTTAATGACCTTATATCTTTTCTATAAAGAAAA

676/74_(34) AAAGTAGTCAATTACATTAAGGTCAAGTTTAATGACCTTATATCTTTTCTATAAAGAAAA

Fiji_(39) ----------ATTACATTAAGGTCAAGTTTAATGACCTTATATCTTTTCTATAAAGAAAA

PMP1351_(33F-1) AAAGTAGTCAATTACATTAAGGTCAAGTTTAATGGCCTTATATCTTTTCTATAAAGAAAA

PMP1352_(33F-1) AAAGTAGTCAATTACATTAAGGTCAAGTTTAATGGCCTTATATCTTTTCTATAAAGAAAA

PMP1379_(33F-1) AAAGTAGTCAATTACATTAAGGTCAAGTTTAATGGCCTTATATCTTTTCTATAAAGAAAA

PMP1380_(33F-1) AAAGTAGTCAATTACATTAAGGTCAAGTTTAATGGCCTTATATCTTTTCTATAAAGAAAA

PMP1386_(33F-1) ------------------------------------------------------------

PMP1387_(33F-1) ----------------TTAAGGTCAAGTTTAATGACCTTATATCTTTTCTATAAAGAAAA

Fiji_(34) ACTACAATTAGAGGAGCGAGATTTAATGTGAAAAAGATAAAAGAATATGATATTTTAAAA

203/40_(39) ACTACAATTAGAGGAGCGAGATTTAATGTGAAAAAGATAAAAGAATATGATATTTTAAAA

676/74_(34) ACTACAATTAGAGGAGCGAGATTTAATGTGAAAAAGATAAAAGAATATGATATTTTAAAA

Fiji_(39) ACTACAATTAGAGGAGCGAGATTTAATGTGAAAAAGATAAAAGAATATGATATTTTAAAA

PMP1351_(33F-1) ACTACAATTAGAGGAGCGAGATTTAATGTGAAAAAGATAAAAGAATATGACATTTTAAAA

PMP1352_(33F-1) ACTACAATTAGAGGAGCGAGATTTAATGTGAAAAAGATAAAAGAATATGACATTTTAAAA

PMP1379_(33F-1) ACTACAATTAGAGGAGCGAGATTTAATGTGAAAAAGATAAAAGAATATGACATTTTAAAA

PMP1380_(33F-1) ACTACAATTAGAGGAGCGAGATTTAATGTGAAAAAGATAAAAGAATATGACATTTTAAAA

PMP1386_(33F-1) ------ATTAGAGGAGCGAGATTTAATGTGAAAAAGATAAAAGAATATGACATTTTAAAA

PMP1387_(33F-1) ACTACAATTAGAGGAGCGAGATTTAATGTGAAAAAGATAAAAGAATATGACATTTTAAAA

******************************************** *********

Fiji_(34) ATTATGGCTATTATTTTAGTTGTGTTAAGTCATAGTGCGTATTATAAAATATCGAGCAAT

203/40_(39) ATTATGGCTATTATTTTAGTTGTGTTAAGTCATAGTGCGTATTATAAAATATCGAGCAAT

676/74_(34) ATTATGGCTATTATTTTAGTTGTGTTAAGTCATAGTGCGTATTATAAAATATCGAGCAAT

Fiji_(39) ATTATGGCTATTATTTTAGTTGTGTTAAGTCATAGTGCGTATTATAAAATATCGAGCAAT

PMP1351_(33F-1) ATTATGGCTATTATTTTAGTTGTGTTAAGTCATAGTGCGTATTATAAAATATCGAGCAAT

PMP1352_(33F-1) ATTATGGCTATTATTTTAGTTGTGTTAAGTCATAGTGCGTATTATAAAATATCGAGCAAT

PMP1379_(33F-1) ATTATGGCTATTATTTTAGTTGTGTTAAGTCATAGTGCGTATTATAAAATATCGAGCAAT

PMP1380_(33F-1) ATTATGGCTATTATTTTAGTTGTGTTAAGTCATAGTGCGTATTATAAAATATCGAGCAAT

PMP1386_(33F-1) ATTATGGCTATTATTTTAGTTGTGTTAAGTCATAGTGCGTATTATAAAATATCGAGCAAT

PMP1387_(33F-1) ATTATGGCTATTATTTTAGTTGTGTTAAGTCATAGTGCGTATTATAAAATATCGAGCAAT

************************************************************

Fiji_(34) TATGGTGGAATGGATTATCAACAATATTTAAATAGTCATTCAGCATTTACTCTATATAAG

203/40_(39) TATGGTGGAATGGATTATCAACAATATTTAAATAGTCATTCAGCATTTACTCTATATAAG

676/74_(34) TATGGTGGAATGGATTATCAACAATATTTAAATAGTCATTCAGCATTTACTCTATATAAG

Fiji_(39) TATGGTGGAATGGATTATCAACAATATTTAAATAGTCATTCAGCATTTACTCTATATAAG

PMP1351_(33F-1) TATGGTGGAATGGATTATCAACAATATTTAAATAGTCATTCAGCATTTACTCTATATGAG

PMP1352_(33F-1) TATGGTGGAATGGATTATCAACAATATTTAAATAGTCATTCAGCATTTACTCTATATGAG

PMP1379_(33F-1) TATGGTGGAATGGATTATCAACAATATTTAAATAGTCATTCAGCATTTACTCTATATGAG

PMP1380_(33F-1) TATGGTGGAATGGATTATCAACAATATTTAAATAGTCATTCAGCATTTACTCTATATGAG

PMP1386_(33F-1) TATGGTGGAATGGATTATCAACAATATTTAAATAGTCATTCAGCATTTACTCTATATGAG

PMP1387_(33F-1) TATGGTGGAATGGATTATCAACAATATTTAAATAGTCATTCAGCATTTACTCTATATGAG

********************************************************* **

Fiji_(34) ATACTTGGTAAATTTATGGAAATTATCTATTATTTTCATATGCCATTATTTATGGCTATA

203/40_(39) ATACTTGGTAAATTTATGGAAATTATCTATTATTTTCATATGCCATTATTTATGGCTATA

676/74_(34) ATACTTGGTAAATTTATGGAAATTATCTATTATTTTCATATGCCATTATTTATGGCTATA

Fiji_(39) ATACTTGGTAAATTTATGGAAATTATCTATTATTTTCATATGCCATTATTTATGGCTATA

PMP1351_(33F-1) ATACTTGGTAAATTTATGGAAATTATCTATTATTTTCATATACCATTATTTATGGCTATA

PMP1352_(33F-1) ATACTTGGTAAATTTATGGAAATTATCTATTATTTTCATATACCATTATTTATGGCTATA

PMP1379_(33F-1) ATACTTGGTAAATTTATGGAAATTATCTATTATTTTCATATACCATTATTTATGGCTATA

PMP1380_(33F-1) ATACTTGGTAAATTTATGGAAATTATCTATTATTTTCATATACCATTATTTATGGCTATA

PMP1386_(33F-1) ATACTTGGTAAATTTATGGAAATTATCTATTATTTTCATATACCATTATTTATGGCTATA

PMP1387_(33F-1) ATACTTGGTAAATTTATGGAAATTATCTATTATTTTCATATACCATTATTTATGGCTATA

***************************************** ******************

Fiji_(34) TCGGGTGTATTTTTCTCTATTCAAATAAAAAAAGATCGATGGAATAAGATTGAGAAATTA

203/40_(39) TCGGGTGTATTTTTCTCTATTCAAATAAAAAAAGATCGATGGAATAAGATTGAGAAATTA

676/74_(34) TCGGGTGTATTTTTCTCTATTCAAATAAAAAAAGATCGATGGAATAAGATTGAGAAATTA

Fiji_(39) TCGGGTGTATTTTTCTCTATTCAAATAAAAAAAGATCGATGGAATAAGATTGAGAAATTA

PMP1351_(33F-1) TCGGGTGTATTTTTCTCTATTCAAATAAAAAAAGATCGATGGAATAAGATTGAGAAATTA

PMP1352_(33F-1) TCGGGTGTATTTTTCTCTATTCAAATAAAAAAAGATCGATGGAATAAGATTGAGAAATTA

PMP1379_(33F-1) TCGGGTGTATTTTTCTCTATTCAAATAAAAAAAGATCGATGGAATAAGATTGAGAAATTA

PMP1380_(33F-1) TCGGGTGTATTTTTCTCTATTCAAATAAAAAAAGATCGATGGAATAAGATTGAGAAATTA

PMP1386_(33F-1) TCGGGTGTATTTTTCTCTATTCAAATAAA-AAAGATCGATGGAATAAGATTGAGAAATTA

PMP1387_(33F-1) TCGGGTGTATTTTTCTCTATTCAAATAAA-AAAGATCGATGGAATAAGATTGAGAAATTA

***************************** ******************************

Fiji_(34) TTAACTAGTAAGTTTAAAAGATTAATATTGCCATTT-TTTGTTTTTACTTTATTATATAG

203/40_(39) TTAACTAGTAAGTTTAAAAGATTAATATTGCCATTT-TTTGTTTTTACTTTATTATATAG

676/74_(34) TTAACTAGTAAGTTTAAAAGATTAATATTGCCATTT-TTTGTTTTTACTTTATTATATAG

Fiji_(39) TTAACTAGTAAGTTTAAAAGATTAATATTGCCATTT-TTTGTTTTTACTTTATTATATAG

PMP1351_(33F-1) TTAACTAGTAAGTTTAAAAGATTAATATTGCCATTTTTTTGTTTTTACTTTATTATATAG

PMP1352_(33F-1) TTAACTAGTAAGTTTAAAAGATTAATATTGCCATTTTTTTGTTTTTACTTTATTATATAG

PMP1379_(33F-1) TTAACTAGTAAGTTTAAAAGATTAATATTGCCATTTTTTTGTTTTTACTTTATTATATAG

PMP1380_(33F-1) TTAACTAGTAAGTTTAAAAGATTAATATTGCCATTTTTTTGTTTTTACTTTATTATATAG

PMP1386_(33F-1) TTAACTAGTAAGTTTAAAAGATTAATATTGCCATTT-TTTGTTTTTACTTTATTATATAG

PMP1387_(33F-1) TTAACTAGTAAGTTTAAAAGATTAATATTGCCATTT-TTTGTTTTTACTTTATTATATAG

************************************ ***********************

Fiji_(34) TTTGCCATTAAAATATATATCAAACTACTACAATGGTGTTTCATTTTGGAGAGCTATAAC

203/40_(39) TTTGCCATTAAAATATATATCAAACTACTACAATGGTGTTTCATTTTGGAGAGCTATAAC

676/74_(34) TTTGCCATTAAAATATATATCAAACTACTACAATGGTGTTTCGTTTTGGAGAGCTATAAC

Fiji_(39) TTTGCCATTAAAATATATATCAAACTACTACAATGGTGTTTCATTTTGGAGAGCTATAAC

PMP1351_(33F-1) TTTGCCATTAAAATATATATCAAACTACTACAATGGTGTTTCATTTTGGAGAGCTATAAC

PMP1352_(33F-1) TTTGCCATTAAAATATATATCAAACTACTACAATGGTGTTTCATTTTGGAGAGCTATAAC

PMP1379_(33F-1) TTTGCCATTAAAATATATATCAAACTACTACAATGGTGTTTCATTTTGGAGAGCTATAAC

PMP1380_(33F-1) TTTGCCATTAAAATATATATCAAACTACTACAATGGTGTTTCATTTTGGAGAGCTATAAC

PMP1386_(33F-1) TTTGCCATTAAAATATATATCAAACTACTACAATGGTGTTTCATTTTGGAGAGCTATAAC

PMP1387_(33F-1) TTTGCCATTAAAATATATATCAAACTACTACAATGGTGTTTCATTTTGGAGAGCTATAAC

****************************************** *****************

Fiji_(34) TGGTCAATTCTTGTTATTAGGAAATTCTCACTTATGGTATTTGTATGCATTATTTATTAT

203/40_(39) TGGTCAATTCTTGTTATTAGGAAATTCTCACTTATGGTATTTGTATGCATTATTTATTAT

676/74_(34) TGGTCAATTCTTGTTATTAGGAAATTCTCACTTATGGTATTTGTATGCATTATTTATTAT

Fiji_(39) TGGTCAATTCTTGTTATTAGGAAATTCTCACTTATGGTATTTGTATGCATTATTTATTAT

PMP1351_(33F-1) TGGTCAATTCTTGTTATTAGGAAATTCTCACTTATGGTATTTGTATGCATTATTTATTAT

PMP1352_(33F-1) TGGTCAATTCTTGTTATTAGGAAATTCTCACTTATGGTATTTGTATGCATTATTTATTAT

PMP1379_(33F-1) TGGTCAATTCTTGTTATTAGGAAATTCTCACTTATGGTATTTGTATGCATTATTTATTAT

PMP1380_(33F-1) TGGTCAATTCTTGTTATTAGGAAATTCTCACTTATGGTATTTGTATGCATTATTTATTAT

PMP1386_(33F-1) TGGTCAATTCTTGTTATTAGGAAATTCTCACTTATGGTATTTGTATGCATTATTTATTAT

PMP1387_(33F-1) TGGTCAATTCTTGTTATTAGGAAATTCTCACTTATGGTATTTGTATGCATTATTTATTAT

************************************************************

Fiji_(34) CTTTATAATTAGTTTTTATTGTTTAAGAAGAGATACTTCTATATTTGTATACTTGTCCTT

203/40_(39) CTTTATAATTAGTTTTTATTGTTTAAGAAGAGATACTTCTATATTTGTATACTTGTCCTT

676/74_(34) CTTTATAATTAGTTTTTATTGTTTAAGAAGAGATACTTCTATATTTGTATACTTGTCCTT

Fiji_(39) CTTTATAATTAGTTTTTATTGTTTAAGAAGAGATACTTCTATATTTGTATACTTGTCCTT

PMP1351_(33F-1) CTTTATAATTAGTTTTTATTGTTTAAGAAGAGATACTTCTATATTTGTATACTTGTCCTT

PMP1352_(33F-1) CTTTATAATTAGTTTTTATTGTTTAAGAAGAGATACTTCTATATTTGTATACTTGTCCTT

PMP1379_(33F-1) CTTTATAATTAGTTTTTATTGTTTAAGAAGAGATACTTCTATATTTGTATACTTGTCCTT

PMP1380_(33F-1) CTTTATAATTAGTTTTTATTGTTTAAGAAGAGATACTTCTATATTTGTATACTTGTCCTT

PMP1386_(33F-1) CTTTATAATTAGTTTTTATTGTTTAAGAAGAGATACTTCTATATTTGTATACTTGTCCTT

PMP1387_(33F-1) CTTTATAATTAGTTTTTATTGTTTAAGAAGAGATACTTCTATATTTGTATACTTGTCCTT

************************************************************

Fiji_(34) ATATATTATACATGTACTGAGTTTCTTGATTCATATAACGTTAGTAAGTGCACCATTGCA

203/40_(39) ATATATTATACATGTACTGAGTTTCTTGATTCATATAACGTTAGTAAGTGCACCATTGCA

676/74_(34) ATATATTATACATGTACTGAGTTTCTTGATTCATATAACGTTAGTAAGTGCACCATTGCA

Fiji_(39) ATATATTATACATGTACTGAGTTTCTTGATTCATATAACGTTAGTAAGTGCACCATTGCA

PMP1351_(33F-1) ATATATTATACATGTACTGAGTTTCTTGATTCATATAACGTTAGTAAGTGCACCATTGCA

PMP1352_(33F-1) ATATATTATACATGTACTGAGTTTCTTGATTCATATAACGTTAGTAAGTGCACCATTGCA

PMP1379_(33F-1) ATATATTATACATGTACTGAGTTTCTTGATTCATATAACGTTAGTAAGTGCACCATTGCA

PMP1380_(33F-1) ATATATTATACATGTACTGAGTTTCTTGATTCATATAACGTTAGTAAGTGCACCATTGCA

PMP1386_(33F-1) ATATATTATACATGTACTGAGTTTCTTGATTCATATAACGTTAGTAAGTGCACCATTGCA

PMP1387_(33F-1) ATATATTATACATGTACTGAGTTTCTTGATTCATATAACGTTAGTAAGTGCACCATTGCA

************************************************************

Fiji_(34) GTTTCTTTTTTGGTTTTCCATGGGATTTTTGTTCGAATCTAAAAGAAGGAAGTATAATAT

203/40_(39) GTTTCTTTTTTGGTTTTCCATGGGATTTTTGTTCGAATCTAAAAGAAGGAAGTATAATAT

676/74_(34) GTTTCTTTTTTGGTTTTCCATGGGATTTTTGTTCGAATCTAAAAGAAGGAAGTATAATAT

Fiji_(39) GTTTCTTTTTTGGTTTTCCATGGGATTTTTGTTCGAATCTAAAAGAAGGAAGTATAATAT

PMP1351_(33F-1) GTTTCTTTTTTGGTTTTCCATGGGATTTTTGTTCGAATCTAAAAGAAGGAAGTATAATAT

PMP1352_(33F-1) GTTTCTTTTTTGGTTTTCCATGGGATTTTTGTTCGAATCTAAAAGAAGGAAGTATAATAT

PMP1379_(33F-1) GTTTCTTTTTTGGTTTTCCATGGGATTTTTGTTCGAATCTAAAAGAAGGAAGTATAATAT

PMP1380_(33F-1) GTTTCTTTTTTGGTTTTCCATGGGATTTTTGTTCGAATCTAAAAGAAGGAAGTATAATAT

PMP1386_(33F-1) GTTTCTTTTTTGGTTTTCCATGGGATTTTTGTTCGAATCTAAAAGAAGGAAGTATAATAT

PMP1387_(33F-1) GTTTCTTTTTTGGTTTTCCATGGGATTTTTGTTCGAATCTAAAAGAAGGAAGTATAATAT

************************************************************

Fiji_(34) TTTTTTGGAAAATCACAAATGGATTAGCCTTTTATTCTTTGTGTTATTTATATTTTTGGT

203/40_(39) TTTTTTGGAAAATCACAAATGGATTAGCCTTTTATTCTTTGTGTTATTTATATTTTTGGT

676/74_(34) TTTTTTGGAAAATCACAAATGGATTAGCCTTTTATTCTTTGTGTTATTTATATTTTTGGT

Fiji_(39) TTTTTTGGAAAATCACAAATGGATTAGCCTTTTATTCTTTGTGTTATTTATATTTTTGGT

PMP1351_(33F-1) TTTTTTGGAAAATCACAAATGGATTAGCCTTTTATTCTTTGTGTTATTTATATTTTTGGT

PMP1352_(33F-1) TTTTTTGGAAAATCACAAATGGATTAGCCTTTTATTCTTTGTGTTATTTATATTTTTGGT

PMP1379_(33F-1) TTTTTTGGAAAATCACAAATGGATTAGCCTTTTATTCTTTGTGTTATTTATATTTTTGGT

PMP1380_(33F-1) TTTTTTGGAAAATCACAAATGGATTAGCCTTTTATTCTTTGTGTTATTTATATTTTTGGT

PMP1386_(33F-1) TTTTTTGGAAAATCACAAATGGATTAGCCTTTTATTCTTTGTGTTATTTATATTTTTGGT

PMP1387_(33F-1) TTTTTTGGAAAATCACAAATGGATTAGCCTTTTATTCTTTGTGTTATTTATATTTTTGGT

************************************************************

Fiji_(34) GGTTTTAAATTTTCTATTTAAAAGCGATTTTAAAGTATTGAGTCGATTTTTTGTTGACTT

203/40_(39) GGTTTTAAATTTTCTATTTAAAAGCGATTTTAAAGTATTGAGTCGATTTTTTGTTGACTT

676/74_(34) GGTTTTAAATTTTCTATTTAAAAGCGATTTTAAAGTATTGAGTCGATTTTTTGTTGACTT

Fiji_(39) GGTTTTAAATTTTCTATTTAAAAGCGATTTTAAAGTATTGAGTCGATTTTTTGTTGACTT

PMP1351_(33F-1) GGTTTTAAATTTTCTATTTAAAAGCGATTTTAAAGTATTGAGTCGATTTTTTGTTGACTT

PMP1352_(33F-1) GGTTTTAAATTTTCTATTTAAAAGCGATTTTAAAGTATTGAGTCGATTTTTTGTTGACTT

PMP1379_(33F-1) GGTTTTAAATTTTCTATTTAAAAGCGATTTTAAAGTATTGAGTCGATTTTTTGTTGACTT

PMP1380_(33F-1) GGTTTTAAATTTTCTATTTAAAAGCGATTTTAAAGTATTGAGTCGATTTTTTGTTGACTT

PMP1386_(33F-1) GGTTTTAAATTTTCTATTTAAAAGCGATTTTAAAGTATTGAGTCGATTTTTTGTTGACTT

PMP1387_(33F-1) GGTTTTAAATTTTCTATTTAAAAGCGATTTTAAAGTATTGAGTCGATTTTTTGTTGACTT

************************************************************

Fiji_(34) ATTAGCTATTTTAGGCTCACTTATTTGTTACAATATTTCGTACTTTTTAAGTAATAAAAC

203/40_(39) ATTAGCTATTTTAGGATCACTTATTTGTTACAATATTTCGTACTTTTTAAGTAATAAAAC

676/74_(34) ATTAGCTATTTTAGGATCACTTATTTGTTACAATATTTCGTACTTTTTAAGTAATAAAAC

Fiji_(39) ATTAGCTATTTTAGGATCACTTATTTGTTACAATATTTCGTACTTTTTAAGTAATAAAAC

PMP1351_(33F-1) ATTAGCTATTTTAGGATCACTTATTTGTTACAATATTTCGTACTTTTTAAGTAATAAAAC

PMP1352_(33F-1) ATTAGCTATTTTAGGATCACTTATTTGTTACAATATTTCGTACTTTTTAAGTAATAAAAC

PMP1379_(33F-1) ATTAGCTATTTTAGGATCACTTATTTGTTACAATATTTCGTACTTTTTAAGTAATAAAAC

PMP1380_(33F-1) ATTAGCTATTTTAGGATCACTTATTTGTTACAATATTTCGTACTTTTTAAGTAATAAAAC

PMP1386_(33F-1) ATTAGCTATTTTAGGATCACTTATTTGTTACAATATTTCGTACTTTTTAAGTAATAAAAC

PMP1387_(33F-1) ATTAGCTATTTTAGGATCACTTATTTGTTACAATATTTCGTACTTTTTAAGTAATAAAAC

*************** ********************************************

Fiji_(34) TAAAATTTTAGATAGTAAACTACTTAATCTCATTTTGATTAATGGTTTAGGGATATATAT

203/40_(39) TAAAATTTTAGATAGTAAACTACTTAATCTCATTTTGATTAATGGTTTAGGGATATATAT

676/74_(34) TAAAATTTTAGATAGTAAACTACTTAATCTCATTTTGATTAATGGTTTAGGGATATATAT

Fiji_(39) TAAAATTTTAGATAGTAAACTACTTAATCTCATTTTGATTAATGGTTTAGGGATATATAT

PMP1351_(33F-1) TAAAATTTTAGATAGTAAACTACTTAATCTCATTTTGATTAATGCTTTAGGGATATATAT

PMP1352_(33F-1) TAAAATTTTAGATAGTAAACTACTTAATCTCATTTTGATTAATGCTTTAGGGATATATAT

PMP1379_(33F-1) TAAAATTTTAGATAGTAAACTACTTAATCTCATTTTGATTAATGCTTTAGGGATATATAT

PMP1380_(33F-1) TAAAATTTTAGATAGTAAACTACTTAATCTCATTTTGATTAATGCTTTAGGGATATATAT

PMP1386_(33F-1) TAAAATTTTAGATAGTAAACTACTTAATCTCATTTTGATTAATGCTTTAGGGATATATAT

PMP1387_(33F-1) TAAAATTTTAGATAGTAAACTACTTAATCTCATTTTGATTAATGCTTTAGGGATATATAT

******************************************** ***************

Fiji_(34) TTTTTCTGATACTTTAAACTATTTTATATTAAGTATTTCTTATTTTGTAAGTGATAGATT

203/40_(39) TTTTTCTGATACTTTAAACTATTTTATATTAAGTATTTCTTATTTTGTAAGTGATAGATT

676/74_(34) TTTTTCTGATACTTTAAACTATTTTATATTAAGTATTTCTTATTTTGTAAGTGATAGATT

Fiji_(39) TTTTTCTGATACTTTAAACTATTTTATATTAAGTATTTCTTATTTTGTAAGTGATAGATT

PMP1351_(33F-1) TTTTTCTGATACTTTAAACTATTTTATATTAAGTATTTCTTATTTTGTAAGTGATAGATT

PMP1352_(33F-1) TTTTTCTGATACTTTAAACTATTTTATATTAAGTATTTCTTATTTTGTAAGTGATAGATT

PMP1379_(33F-1) TTTTTCTGATACTTTAAACTATTTTATATTAAGTATTTCTTATTTTGTAAGTGATAGATT

PMP1380_(33F-1) TTTTTCTGATACTTTAAACTATTTTATATTAAGTATTTCTTATTTTGTAAGTGATAGATT

PMP1386_(33F-1) TTTTTCTGATACTTTAAACTATTTTATATTAAGTATTTCTTATTTTGTAAGTGATAGATT

PMP1387_(33F-1) TTTTTCTGATACTTTAAACTATTTTATATTAAGTATTTCTTATTTTGTAAGTGATAGATT

************************************************************

Fiji_(34) TATGTTTACTTCATTTGGTATAATTATTATATTTTTAATTAGATTTGTTTTCACTTTATT

203/40_(39) TATGTTTACTTCATTTGGTATAATTATTATATTTTTAATTAGATTTGTTTTCACTTTATT

676/74_(34) TATGTTTACTTCATTTGGTATAATTATTATATTTTTAATTAGATTTGTTTTCACTTTATT

Fiji_(39) TATGTTTACTTCATTTGGTATAATTATTATATTTTTAATTAGATTTGTTTTCACTTTATT

PMP1351_(33F-1) TATGTTTACTTCATTTGGTATAATTATTATATTTTTAATTAGATTTGTTTTCACTTTATT

PMP1352_(33F-1) TATGTTTACTTCATTTGGTATAATTATTATATTTTTAATTAGATTTGTTTTCACTTTATT

PMP1379_(33F-1) TATGTTTACTTCATTTGGTATAATTATTATATTTTTAATTAGATTTGTTTTCACTTTATT

PMP1380_(33F-1) TATGTTTACTTCATTTGGTATAATTATTATATTTTTAATTAGATTTGTTTTCACTTTATT

PMP1386_(33F-1) TATGTTTACTTCATTTGGTATAATTATTATATTTTTAATTAGATTTGTTTTCACTTTATT

PMP1387_(33F-1) TATGTTTACTTCATTTGGTATAATTATTATATTTTTAATTAGATTTGTTTTCACTTTATT

************************************************************

Fiji_(34) TTTGGGATTAGTCTTTACATTGCTATTTAAAAAAGTATTTCCAAAATATTCTTGGTTAGT

203/40_(39) TTTGGGATTAGTCTTTACATTGCTATTTAAAAAAGTATTTCCAAAATATTCTTGGCTAGT

676/74_(34) TTTGGGATTAGTCTTTACATTGCTATTTAAAAAAGTATTTCCAAAATATTCTTGGTTAGT

Fiji_(39) TTTGGGATTAGTCTTTACATTGCTATTTAAAAAAGTATTTCCAAAATATTCTTGGTTAGT

PMP1351_(33F-1) TTTGGGATTAGTCTTTACATTGCTATTTAAAAAAGTATTTCCAAAATATTCTTGGTTAGT

PMP1352_(33F-1) TTTGGGATTAGTCTTTACATTGCTATTTAAAAAAGTATTTCCAAAATATTCTTGGTTAGT

PMP1379_(33F-1) TTTGGGATTAGTCTTTACATTGCTATTTAAAAAAGTATTTCCAAAATATTCTTGGTTAGT

PMP1380_(33F-1) TTTGGGATTAGTCTTTACATTGCTATTTAAAAAAGTATTTCCAAAATATTCTTGGTTAGT

PMP1386_(33F-1) TTTGGGATTAGTCTTTACATTGCTATTTAAAAAAGTATTTCCAAAATATTCTTGGTTAGT

PMP1387_(33F-1) TTTGGGATTAGTCTTTACATTGCTATTTAAAAAAGTATTTCCAAAATATTCTTGGTTAGT

******************************************************* ****

Fiji_(34) TAACTAGAAAATACAATATCAGTGATTATGCACTATAAGCTATCCTGTAATTCTTACAGT

203/40_(39) TAACTAGAAAATACAATATCAGTGATTATGCACTATAAGCTATCCTGTAATTCTTACAGT

676/74_(34) TAACTAGAAAATACAATATCAGTGATTATGCACTATAAGCTATCCTGTAATTCTTACAGT

Fiji_(39) TAACTAGAAAATACAATATCAGTGATTATGCACTATAAGCTATCCTGTAATTCTTACAGT

PMP1351_(33F-1) TAACTAGAAAATACAATATCAGTGATTATGCACTATAAGCTATCCTGTAATTCTTACAGT

PMP1352_(33F-1) TAACTAGAAAATACAATATCAGTGATTATGCACTATAAGCTATCCTGTAATTCTTACAGT

PMP1379_(33F-1) TAACTAGAAAATACAATATCAGTGATTATGCACTATAAGCTATCCTGTAATTCTTACAGT

PMP1380_(33F-1) TAACTAGAAAATACAATATCAGTGATTATGCACTATAAGCTATCCTGTAATTCTTACAGT

PMP1386_(33F-1) TAACTAGAAAATACAATATCAGTGATTATGCACTATAAGCTATCCTGTAATTCTTACAGT

PMP1387_(33F-1) TAACTAGAAAATACAATATCAGTGATTATGCACTATAAGCTATCCTGTAATTCTTACAGT

************************************************************

Fiji_(34) TAAATATTTTATCCTATATACTGAGGAGACAAATAAAATAACTTATAGTATTTTGCACCT

203/40_(39) TAAATATTTTATCCTATATACTGAGGAGACAAATAAAATAACTTATAGTATTTTGCACCT

676/74_(34) TAAATATTTTATCCTATATACTGAGGAGACAAATAAAATAACTTATAGTATTTTGCACCT

Fiji_(39) TAAATATTTTATCCTATATACTGAGGAGACAAATAAAATAACTTATAGTATTTTGCACCT

PMP1351_(33F-1) TAAATATTTTATCCTATATACTGAGGAGACAAATAAAATAACTTATAGTATTTTGCACCT

PMP1352_(33F-1) TAAATATTTTATCCTATATACTGAGGAGACAAATAAAATAACTTATAGTATTTTGCACCT

PMP1379_(33F-1) TAAATATTTTATCCTATATACTGAGGAGACAAATAAAATAACTTATAGTATTTTGCACCT

PMP1380_(33F-1) TAAATATTTTATCCTATATACTGAGGAGACAAATAAAATAACTTATAGTATTTTGCACCT

PMP1386_(33F-1) TAAATATTTTATCCTATATACTGAGGAGACAAATAAAATAACTTATAGTATTTTGCACCT

PMP1387_(33F-1) TAAATATTTTATCCTATATACTGAGGAGACAAATAAAATAACTTATAGTATTTTGCACCT

************************************************************

Fiji_(34) TATCTAGTTTAATTGTAATATAAAAGAAAATATCCAGGAAGTGGTATAGTACTCTCTCAT

203/40_(39) TATCTAGTTTAATTGTAATATAAAAGAAAATATCCAGGAAGTGGTATAGTACTCTCTCAT

676/74_(34) TATCTAGTTTAATTGTAATATAAAAGAAAATATCCAGGAAGTGGTATAGTACTCTCTCAT

Fiji_(39) TATCTAGTTTAATTGTAATATAAAAGAAAATATCCAGGAAGTGGTATAGTACTCTCTCAT

PMP1351_(33F-1) TATCTAGTTTAATTGTAATATAAAAGAAAATATCCAGGAAGTGGTATAGTACTCTCTCAT

PMP1352_(33F-1) TATCTAGTTTAATTGTAATATAAAAGAAAATATCCAGGAAGTGGTATAGTACTCTCTCAT

PMP1379_(33F-1) TATCTAGTTTAATTGTAATATAAAAGAAAATATCCAGGAAGTGGTATAGTACTCTCTCAT

PMP1380_(33F-1) TATCTAGTTTAATTGTAATATAAAAGAAAATATCCAGGAAGTGGTATAGTACTCTCTCAT

PMP1386_(33F-1) TATCTAGTTTAATTGTAATATAAAAGAAAATATCCAGGAAGTGGTATAGTACTCTCTCAT

PMP1387_(33F-1) TATCTAGTTTAATTGTAATATAAAAGAAAATATCCAGGAAGTGGTATAGTACTCTCTCAT

************************************************************

Fiji_(34) TGTTTAACTTAGCTTAAATATATAGGTTAATTCCTTGACTATGTGATATAGTTGAGGGAT

203/40_(39) TGTTTAACTTCGCTTAAATATATAGGTTAATTCCTTGACTATGTGATGTAGTTGAGGGAT

676/74_(34) TGTTTAACTTCGCTTAAATATATAGGTTAATTCCTTGACTATGTGATGTAGTTGAGGGAT

Fiji_(39) TGTTTAACTTCGCTTAAATATATAGGTTAATTCCTTGACTATGTGATGTAGTTGAGGGAT

PMP1351_(33F-1) TGTTTAACTTCGCTTAAATATATAGGTTAATTCCTTGACTATGTGATATAGTTGAGGGAT

PMP1352_(33F-1) TGTTTAACTTCGCTTAAATATATAGGTTAATTCCTTGACTATGTGATATAGTTGAGGGAT

PMP1379_(33F-1) TGTTTAACTTCGCTTAAATATATAGGTTAATTCCTTGACTATGTGATATAGTTGAGGGAT

PMP1380_(33F-1) TGTTTAACTTCGCTTAAATATATAGGTTAATTCCTTGACTATGTGATATAGTTGAGGGAT

PMP1386_(33F-1) TGTTTAACTTCGCTTAAATATATAGGTTAATTCCTTGACTATGTGATATAGTTGAGGGAT

PMP1387_(33F-1) TGTTTAACTTCGCTTAAATATATAGGTTAATTCCTTGACTATGTGATATAGTTGAGGGAT

********** ************************************ ************

Fiji_(34) TTTTAAATGATATTCATATTTTTTGCAAAGATGTTGTT----------------------

203/40_(39) TTTTGATA-ATATTCATATTTTTTGCAAAGATGTTGTTTGAAAAATAATTTTCAAAAATT

676/74_(34) TTTTGATA-ATATTCATATTTTTTGCAAAGATGTTGTTTGAAAAATAATTTTCAAAAATT

Fiji_(39) TTTTGATA-ATATTCATATTTTTTGCAAAGATGTTGTT----------------------

PMP1351_(33F-1) TTTTAAATGATATTCATATTTTTTGCAAAGATGTTG------------------------

PMP1352_(33F-1) TTTTAAATGATATTCATATTTTTTGCAAAGATGTTG------------------------

PMP1379_(33F-1) TTTTAAATGATATTCATATTTTTTGCAAAGATGTTG------------------------

PMP1380_(33F-1) TTTTAAATGATATTCATATTTTTTGCAAAGATGTTG------------------------

PMP1386_(33F-1) TTTTAAATGATATTCATATTTTTTGCAAAGATGTTGTT----------------------

PMP1387_(33F-1) TTTTAAATGATATTCATATTTTTTGCAAAGATGTTGTTTGAAAAATAATTTCAAAAATT-

**** * ***************************

**S2 Fig. Sanger sequencing of 33F-1 *wcyO* region from four 33F-1 isolates (PMP1351, PMP1352, PMP1379, PMP1380, PMP1386, PMP1387).** These sequences were aligned to a representative sequence from serotype 34 strain 676/74 (Genbank accession no. CR931703), serotype 39 strain 203/40 (Genbank accession no. CR931711) and representative *wcyO* sequences from serotype 34 and 39 isolates from Fiji using Clustal Omega. Red sequence indicates the 3’end of *glf,* blue indicates *wcyO,* and the highlighted regions denote the frameshift mutation site in the 33F-1 sequences. Identical nucleotides are denoted by an asterisk.
