# Supplementary material for: Discovery of a Streptococcus pneumoniae serotype 33F capsular polysaccharide locus that lacks wcjE and contains a wcyO pseudogene
Source: PLoS One. 2018 Nov 5;13(11):e0206622. doi: 10.1371/journal.pone.0206622 (PMC6218050; doi:10.1371/journal.pone.0206622)
Supplement: S3 Fig — Identical, conserved and semi-conserved residues are denoted by the symbols ‘*’, ‘:’ and ‘.’, respectively. (DOCX) [file pone.0206622.s005.docx]

**Wzg (Identity: 96.9%, Similarity: 98.5%)**

33F-1_Wzg 1 MSRRFKKSRSQKVKRSVNIVLLTIYLLLVGFLLFLIFKYNILAFRYLNLV 50

|||||||||||||||||||||||||||||.||||||||||||||||||||

33F_Wzg 1 MSRRFKKSRSQKVKRSVNIVLLTIYLLLVCFLLFLIFKYNILAFRYLNLV 50

33F-1_Wzg 51 VTALVLLVALVGLLLIIYKKAEKFTIFLLVFSILVSSVSLFAVQQFVGLT 100

|||||||||||||||||||||||||||||:||||||||||||||||||||

33F_Wzg 51 VTALVLLVALVGLLLIIYKKAEKFTIFLLLFSILVSSVSLFAVQQFVGLT 100

33F-1_Wzg 101 NRLNATSNYSEYSISVAVLADSEIENVTQLTSVTAPTGTDNENIQKLLAD 150

||||||||||||||||||||||||||||||||||||||||||||||||||

33F_Wzg 101 NRLNATSNYSEYSISVAVLADSEIENVTQLTSVTAPTGTDNENIQKLLAD 150

33F-1_Wzg 151 IKSSQNTDLMVNQSSSYLAAYKSLIAGETKAIVLNSVFENIIESEYPDYA 200

|||||||||.||||||||||||||||||||||||||||||||||||||||

33F_Wzg 151 IKSSQNTDLTVNQSSSYLAAYKSLIAGETKAIVLNSVFENIIESEYPDYA 200

33F-1_Wzg 201 SKIKKIYTKGFTKKVEAPKTSKNQSFNIYVSGIDTYGPISSVSRSDVNIL 250

||||||||||||||||||||||||||||||||||||||||||||||||||

33F_Wzg 201 SKIKKIYTKGFTKKVEAPKTSKNQSFNIYVSGIDTYGPISSVSRSDVNIL 250

33F-1_Wzg 251 MTVNRDTKKILLTTTPRDAYVPIADGGNNQKDKLTHAGIYGVDSSIHTLE 300

||||||||||||||||||||||||||||||||||||||||||||||||||

33F_Wzg 251 MTVNRDTKKILLTTTPRDAYVPIADGGNNQKDKLTHAGIYGVDSSIHTLE 300

33F-1_Wzg 301 NLYGVDINYYVRLNFTSFLKMIDLLGGVDVHNDQEFSA-LHGKFHFPVGN 349

||||||||||||||||||||:||||||:||:|||||:| .:||: :|.||

33F_Wzg 301 NLYGVDINYYVRLNFTSFLKLIDLLGGIDVYNDQEFTAHTNGKY-YPAGN 349

33F-1_Wzg 350 VHLDSEQALGFVRERYSLADGDRDRGRNQQKVIVAILQKLTSTEALKNYS 399

||||||||||||||||||||||||||||||||||||||||||||||||||

33F_Wzg 350 VHLDSEQALGFVRERYSLADGDRDRGRNQQKVIVAILQKLTSTEALKNYS 399

33F-1_Wzg 400 TIINSLQDSIQTNMPLETMINLVNAQLESGGNYKVNSQDLKGTGRMDLPS 449

|||||||||||||||||||||||||||||||||||||||||||||.||||

33F_Wzg 400 TIINSLQDSIQTNMPLETMINLVNAQLESGGNYKVNSQDLKGTGRTDLPS 449

33F-1_Wzg 450 YAMPDSNLYVMEIDDSSLAVVKAAIQDVMEGR 481

||||||||||||||||||||||||||||||||

33F_Wzg 450 YAMPDSNLYVMEIDDSSLAVVKAAIQDVMEGR 481

**Wzh (Identity: 97.5%, Similarity: 98.8%)**

33F-1_Wzh 1 MIDIHSHIVFDVDDGPKSREESKALLAESYRQGVRTIVSTSHRRKGMFET 50

||||||||||||||||||||||||||.|||||||||||||||||||||||

33F_Wzh 1 MIDIHSHIVFDVDDGPKSREESKALLTESYRQGVRTIVSTSHRRKGMFET 50

33F-1_Wzh 51 PEEKIAENFLQVREIAKEVASDLIIAYGAEIYYTPDVLDKLEKKRIPTLN 100

|||||||||||||||||||||||:||||||||||||||||||||||||||

33F_Wzh 51 PEEKIAENFLQVREIAKEVASDLVIAYGAEIYYTPDVLDKLEKKRIPTLN 100

33F-1_Wzh 101 DSRYALIEFSMNTPYRDIHSALSKILMLGITPVIAHIERYDALENNEKRV 150

||||||||||||||||||||||||||||||||||||||||||||||||||

33F_Wzh 101 DSRYALIEFSMNTPYRDIHSALSKILMLGITPVIAHIERYDALENNEKRV 150

33F-1_Wzh 151 RELIDMGCYTQVNSSHVLKSKLFGERYKFMKKRAQYFLEQDLVHVIASDM 200

|||||.|||||||||||||.||||||||||||||||||||||||:|||||

33F_Wzh 151 RELIDRGCYTQVNSSHVLKPKLFGERYKFMKKRAQYFLEQDLVHIIASDM 200

33F-1_Wzh 201 HNLDGRPPHMAEAYDLVTQKYGEAKAQELFIDNPRKIIMDQLI 243

|||||||||||||||||||||||||||||||||||||:|||||

33F_Wzh 201 HNLDGRPPHMAEAYDLVTQKYGEAKAQELFIDNPRKIVMDQLI 243

**Wzd (Identity: 97.8%, Similarity: 99.1%)**

33F-1_Wzd 1 MMKEQNMIEIDVFQLFKTLWKRKLIILIVALVTGAGAFAYSTFIVKPEYT 50

||||||.|||||||||||||||||:|||||||||||||||||||||||||

33F_Wzd 1 MMKEQNTIEIDVFQLFKTLWKRKLMILIVALVTGAGAFAYSTFIVKPEYT 50

33F-1_Wzd 51 STTRIYVVNRDQGDKPGLTNQDLQAGSYLVKDYREIILSQDVLEKVATNL 100

||||||||||:||||||||||||||||||||||||||||||.||||||||

33F_Wzd 51 STTRIYVVNRNQGDKPGLTNQDLQAGSYLVKDYREIILSQDALEKVATNL 100

33F-1_Wzd 101 KLDMPAKTLASKVQVTVPADTRIVSISVKDKQPEEASRIANSLREVAAEK 150

||||||||||||||||||||||||||||||||||||||||||||||||||

33F_Wzd 101 KLDMPAKTLASKVQVTVPADTRIVSISVKDKQPEEASRIANSLREVAAEK 150

33F-1_Wzd 151 IVAVTRVSDVTTLEEARPATTPSSPNVRRNSLFGFLGGAVVTVIAVLLIE 200

||||||||||||||||||||||||||||||||||||||||||||||||||

33F_Wzd 151 IVAVTRVSDVTTLEEARPATTPSSPNVRRNSLFGFLGGAVVTVIAVLLIE 200

33F-1_Wzd 201 VLDTRVKRPEDVEDVLKIPLLGLVPDFDKIK 231

:||||||||||||||||||||||||||||||

33F_Wzd 201 LLDTRVKRPEDVEDVLKIPLLGLVPDFDKIK 231

**Wze (Identity: 99.1, Similarity: 99.6)**

33F-1_Wze 1 MPTLEISQAKLDSVKKAEEYYNALCTNLQLSGDGLKVLSITSVKIGEGKS 50

|||||||||||||||||||||||||||||||||||||.||||||||||||

33F_Wze 1 MPTLEISQAKLDSVKKAEEYYNALCTNLQLSGDGLKVFSITSVKIGEGKS 50

33F-1_Wze 51 TTSANIAWAFARAGYKTLLIDGDIRNSVMLGVFKARNKITGLTEFLSGTT 100

||||||||||||||||||||||||||||||||||||||||||||||||||

33F_Wze 51 TTSANIAWAFARAGYKTLLIDGDIRNSVMLGVFKARNKITGLTEFLSGTT 100

33F-1_Wze 101 DLSQGLCDTNIENLFVIQAGSVSPNPTALLQSKNFTTMLETLRKYFDYII 150

||||||||||||||||||||||||||||||||||||||||||||||||||

33F_Wze 101 DLSQGLCDTNIENLFVIQAGSVSPNPTALLQSKNFTTMLETLRKYFDYII 150

33F-1_Wze 151 VDTAPVGVVIDAAIITRNCDASILVTEAGEINRRDIQKAKEQLEHTGKPF 200

||||||||||||||||||||||||||||||||||||||||||||||||||

33F_Wze 151 VDTAPVGVVIDAAIITRNCDASILVTEAGEINRRDIQKAKEQLEHTGKPF 200

33F-1_Wze 201 LGIVLNKFDTSVNKYGSYGNYGDYGKNKK 229

||||||||||||:||||||||||||||||

33F_Wze 201 LGIVLNKFDTSVDKYGSYGNYGDYGKNKK 229

**WchA (Identity: 97.1%, Similarity: 98.5%)**

33F-1_WchA 1 MNGKILRSSLVIIQSFLVILLTYLLSTVREAEIVSTTAIALYILHYFVFY 50

|||||::.||.|||||||||||||||.|||||||||||||||||||||||

33F_WchA 1 MNGKIVKPSLAIIQSFLVILLTYLLSAVREAEIVSTTAIALYILHYFVFY 50

33F-1_WchA 51 ISDYGQDFFKRGYLIELLQTLKYILFFALAISISNFFLEDRFSISRRGMI 100

||.||||||||||||||:||||||||||||||||||||||||||||||||

33F_WchA 51 ISVYGQDFFKRGYLIELVQTLKYILFFALAISISNFFLEDRFSISRRGMI 100

33F-1_WchA 101 YFLTLHALLVYVLNLFIKWYWKRAYPNFKGSKKILLLTATSRVEKVLDRL 150

|||||||||||||||||||||||.||||||||||||||||.|||||||||

33F_WchA 101 YFLTLHALLVYVLNLFIKWYWKRTYPNFKGSKKILLLTATFRVEKVLDRL 150

33F-1_WchA 151 IESNEVVGKLVAVSVLDKPDFQHDCLKVVAEGEIVNFATHEVVDEVFINL 200

||||||||:|||||||||||||||||||||||||||||||||||||||||

33F_WchA 151 IESNEVVGELVAVSVLDKPDFQHDCLKVVAEGEIVNFATHEVVDEVFINL 200

33F-1_WchA 201 PSEKYNIGELVSQFETMGIDVTVNLNAFDRSLARNKQIREMAGLNVVTFS 250

||||||||||||||||||||||||||||||||||||||||||||||||||

33F_WchA 201 PSEKYNIGELVSQFETMGIDVTVNLNAFDRSLARNKQIREMAGLNVVTFS 250

33F-1_WchA 251 TAFYKTSHVIAKRIIDIVGALVGLILCGLVSIVLVPLIRKDGGSAIFAQT 300

|.|||||||||||||||:||||||||||||||||||||||||||||||||

33F_WchA 251 TTFYKTSHVIAKRIIDIMGALVGLILCGLVSIVLVPLIRKDGGSAIFAQT 300

33F-1_WchA 301 RIGKNGRQFTFYKFRSMCVDAEAKKRELMEQNTMQGGMFKVDDDPRITKI 350

||||||||||||||||||||||||||||||||||||||||||||||||||

33F_WchA 301 RIGKNGRQFTFYKFRSMCVDAEAKKRELMEQNTMQGGMFKVDDDPRITKI 350

33F-1_WchA 351 GRFIRKTSLDELPQFYNVLKGDMSLVGTRPPTVDEYEHYTPEQKRRLSFK 400

||||||||||||||||||||||||||||||||||||||||||||||||||

33F_WchA 351 GRFIRKTSLDELPQFYNVLKGDMSLVGTRPPTVDEYEHYTPEQKRRLSFK 400

33F-1_WchA 401 PGITGLWQVSGRSEIKNFDEVVKLDVAYIDDWTIWKDIEILLKTVKVVLM 450

||:|||||||||||||||||||||||||||||||||||||||||||||||

33F_WchA 401 PGVTGLWQVSGRSEIKNFDEVVKLDVAYIDDWTIWKDIEILLKTVKVVLM 450

33F-1_WchA 451 KDGAK 455

|||||

33F_WchA 451 KDGAK 455

**WciB (Identity: 99.3%, Similarity: 100%)**

33F-1_WciB 1 MERSRLIDVKIIVATHKEVKMPQDNSLYLPIHVGRDGKSDIGFIGDNTGD 50

||||||||||||||||||||||||||||||||||||||||||||||||||

33F_WciB 1 MERSRLIDVKIIVATHKEVKMPQDNSLYLPIHVGRDGKSDIGFIGDNTGD 50

33F-1_WciB 51 NISSLNPYYCELTGLYWAWKNLDYNYLGLVHYRRYFTNKSQGYNENVNMD 100

||||||||||||||||||||||||||||||||||||||||||||||||||

33F_WciB 51 NISSLNPYYCELTGLYWAWKNLDYNYLGLVHYRRYFTNKSQGYNENVNMD 100

33F-1_WciB 101 DVILSRANVEILLEKSDIIVPKKRKYYIETLYSHYAHTLNGEHLDLARKI 150

|:||||||||||||||||||||||||||||||||||||||||||||||||

33F_WciB 101 DLILSRANVEILLEKSDIIVPKKRKYYIETLYSHYAHTLNGEHLDLARKI 150

33F-1_WciB 151 IEQNSSEYLSSFDKVMKQRSGYMFNMFIMKKELLDDYLPWLFSILDTMYE 200

||||||||||||||||||||||||||||||||||||||||||||||||||

33F_WciB 151 IEQNSSEYLSSFDKVMKQRSGYMFNMFIMKKELLDDYLPWLFSILDTMYE 200

33F-1_WciB 201 QMDLTDYTLFESRLFGRVSELLFNVWLCKKGITPKEVPFMYMERVDLFEK 250

||||||||||||||||||||||||||||:|||||||||||||||||||||

33F_WciB 201 QMDLTDYTLFESRLFGRVSELLFNVWLCQKGITPKEVPFMYMERVDLFEK 250

33F-1_WciB 251 GKSFLMAKFFGKKYGQSF 268

||||||||||||||||||

33F_WciB 251 GKSFLMAKFFGKKYGQSF 268

**WciC (Identity: 99.1%, Similarity: 99.4%)**

33F-1_WciC 1 MKVTIIGQIKNKRTGLGKAINDFRDYCCNRATRVTEIDITNNFNFLSSLF 50

||||||||||||||||||||||||||||||||||||||||||||||||||

33F_WciC 1 MKVTIIGQIKNKRTGLGKAINDFRDYCCNRATRVTEIDITNNFNFLSSLF 50

33F-1_WciC 51 QILISDTDVYYFTPAGSVAGNIRDSLFLFFMIMKRKKIVTHFHNSAFGNV 100

||||||||||||||||||||||||||||||||||||||||||||||||||

33F_WciC 51 QILISDTDVYYFTPAGSVAGNIRDSLFLFFMIMKRKKIVTHFHNSAFGNV 100

33F-1_WciC 101 MRQHPTLMIINRILYSKVDLIILLGEKSKIMFQQLRILDEKFKIIRNGVD 150

||||||||||||||||||||||||||||||||||||||||||||||||||

33F_WciC 101 MRQHPTLMIINRILYSKVDLIILLGEKSKIMFQQLRILDEKFKIIRNGVD 150

33F-1_WciC 151 GYLFIEKNELNKKMSDLPINIIFFSNMIREKGYEILLEVAKKMREDEKYH 200

|||||||||||||||||||||||||||||||||||||||||||..|||||

33F_WciC 151 GYLFIEKNELNKKMSDLPINIIFFSNMIREKGYEILLEVAKKMVGDEKYH 200

33F-1_WciC 201 FYFSGKFQDNNLKTRFINEIYSMNNVTYLDGVYGSDKKKLLQKMHYFVLP 250

||||||||||||||||||||||||||||||||||||||||||||||||||

33F_WciC 201 FYFSGKFQDNNLKTRFINEIYSMNNVTYLDGVYGSDKKKLLQKMHYFVLP 250

33F-1_WciC 251 SYYKDETLPISMLEAMANGLYIIVSDVGVVSEVINKETASLIEMINEETA 300

||||||||||||||||||||||||||||||||||||||||||||||||||

33F_WciC 251 SYYKDETLPISMLEAMANGLYIIVSDVGVVSEVINKETASLIEMINEETA 300

33F-1_WciC 301 DSIIEIIDQTSNKLNELDFNVSKYKQELLNENIQASIYQQLERIAN 346

|||||||:||||||||||||||||||||||||||||||||||||||

33F_WciC 301 DSIIEIINQTSNKLNELDFNVSKYKQELLNENIQASIYQQLERIAN 346

**WciD (Identity: 97.9%, Similarity: 99.7%)**

33F-1_WciD 1 LTEKKNTGKILTVVVPSYNAENYLQETMPTILSAKNIERVELLIVNDGST 50

||:|||||||||||||||||||||||||||||||||||||||||||||||

33F_WciD 1 LTKKKNTGKILTVVVPSYNAENYLQETMPTILSAKNIERVELLIVNDGST 50

33F-1_WciD 51 DKTEEIARQFEREYEGIVRVISKENGGHGSAVNAGIENAVGNYFKVVDAD 100

|:|||||||||||||||||||||||.||||||||||||||||||||||||

33F_WciD 51 DRTEEIARQFEREYEGIVRVISKENCGHGSAVNAGIENAVGNYFKVVDAD 100

33F-1_WciD 101 DWVNTNNLEDLIVFLSEVDVDQVLSPYDKIFVNYRGDIEREEECNEFSQV 150

||||||||||||||||||||||||||||||||||||||||||||||||||

33F_WciD 101 DWVNTNNLEDLIVFLSEVDVDQVLSPYDKIFVNYRGDIEREEECNEFSQV 150

33F-1_WciD 151 ENEVIYSAEEFYTRIKQTVGMHSITVKTSLLQENNIRLSEKMFYVDMEYI 200

||||||||||||||||||||||||||||||||||||||||||||||||||

33F_WciD 151 ENEVIYSAEEFYTRIKQTVGMHSITVKTSLLQENNIRLSEKMFYVDMEYI 200

33F-1_WciD 201 VYILPYVKRVVLFDKSIYRYRLGTETQSVSMASYIKNRDMHKQVIYHLVD 250

||:|||||:|||||||||||||||||||:|||||||||||||||||||||

33F_WciD 201 VYMLPYVKKVVLFDKSIYRYRLGTETQSISMASYIKNRDMHKQVIYHLVD 250

33F-1_WciD 251 FYNQMRSSAVLKRITWKLILNLIRQQWIIYFNLSKKEGKNSECFEFDNWL 300

|||||||||||:||||||||||||||||||||||||||||||||||||||

33F_WciD 251 FYNQMRSSAVLRRITWKLILNLIRQQWIIYFNLSKKEGKNSECFEFDNWL 300

33F-1_WciD 301 IKEGRIKKIPLYFFKAVKYIRFKVKYFLGIRK 332

||||||||||||||||||||||||||||||||

33F_WciD 301 IKEGRIKKIPLYFFKAVKYIRFKVKYFLGIRK 332

**WciE (Identity: 99.1%, Similarity 99.7%)**

33F_WciE 1 MRKIGKVINEYFVLRKSFTPAIARNKLFEKFWGRIGNYKIFNNIASNFYQ 50

||||||||||||.|||||||||||||||||||||||||||||||||:|||

33F-1_WciE 1 MRKIGKVINEYFALRKSFTPAIARNKLFEKFWGRIGNYKIFNNIASDFYQ 50

33F_WciE 51 YKHETIINFLEKDFSQFLKSYNFKEVSHKEIEQRKIFSMWIQGYESAPKL 100

||||||||||||||||||||||||||||||||||||||||||||||||||

33F-1_WciE 51 YKHETIINFLEKDFSQFLKSYNFKEVSHKEIEQRKIFSMWIQGYESAPKL 100

33F_WciE 101 VQKTIDSQRKYAEKYGYKFVFLDENNIREYVTLPSEIVEKYENGTIDFIK 150

|||||||||||||||||||||||:||||||||||||||||||||||||||

33F-1_WciE 101 VQKTIDSQRKYAEKYGYKFVFLDKNNIREYVTLPSEIVEKYENGTIDFIK 150

33F_WciE 151 YSDVVRGTLLSKYGGVWLDSTIYVDSSRELNYLKKDFYTIRAKTHERVPK 200

||||||||||||||||||||||||||||||||||||||||||||||||||

33F-1_WciE 151 YSDVVRGTLLSKYGGVWLDSTIYVDSSRELNYLKKDFYTIRAKTHERVPK 200

33F_WciE 201 YIANGRWSAFCLSGEKQNIVFDFLEKFHVAYFMKYDIVLDYFLIDYIIEL 250

||||||||||||||||||||||||||||||||||||||||||||||||||

33F-1_WciE 201 YIANGRWSAFCLSGEKQNIVFDFLEKFHVAYFMKYDIVLDYFLIDYIIEL 250

33F_WciE 251 GYRTNDLIRNYIDKVEENNQELFFLADNFSNQYDEKEWAGVLSTTALFKC 300

||||||||||||||||||||||||||||||||||||||||||||||||||

33F-1_WciE 251 GYRTNDLIRNYIDKVEENNQELFFLADNFSNQYDEKEWAGVLSTTALFKC 300

33F_WciE 301 SYKCPINEATGTYFDRLMKGEL 322

||||||||||||||||||||||

33F-1_WciE 301 SYKCPINEATGTYFDRLMKGEL 322

**WciF (Identity: 98.5%, Similarity: 99.1%)**

33F-1_WciF 1 MISVIVPVYNVADYLRFALDSLLEQTYKDFEIILVNDGSTDNSGEICDEY 50

|||||||||||||||||||||||||||||||:||||||||||||||||||

33F_WciF 1 MISVIVPVYNVADYLRFALDSLLEQTYKDFEVILVNDGSTDNSGEICDEY 50

33F-1_WciF 51 GKLYDNIYVFHKKNGGLSDARNFGLEKSRGEFITFLDSDDYFEPYALELL 100

|||||||:||||||||||||||||||||||||||||||||||||||||||

33F_WciF 51 GKLYDNIHVFHKKNGGLSDARNFGLEKSRGEFITFLDSDDYFEPYALELL 100

33F-1_WciF 101 ITIQKKYDVDIVSTKGVITYSHDIYSKKLMDEDYLTVKILTNKEFLAAAY 150

||||||||||||||||.|||||||||||||.|||||||||||||||||.|

33F_WciF 101 ITIQKKYDVDIVSTKGGITYSHDIYSKKLMAEDYLTVKILTNKEFLAAVY 150

33F-1_WciF 151 YNDEMTVSAWGKLYKRDLFKTIFPKGKIYEDLYVVAERLLNIKTVAHTDL 200

||||||||||||||||||||||||||||||||||||||||||||||||||

33F_WciF 151 YNDEMTVSAWGKLYKRDLFKTIFPKGKIYEDLYVVAERLLNIKTVAHTDL 200

33F-1_WciF 201 PIYHYYQRQGSIVNSTFSDRQYDFFDAIDHNEAIIKKFYCGDKELLAALN 250

||||||||||||||||||||||||||||||||||||||||||||||||||

33F_WciF 201 PIYHYYQRQGSIVNSTFSDRQYDFFDAIDHNEAIIKKFYCGDKELLAALN 250

33F-1_WciF 251 AKRVIGSFILSNSAFYNSKNDITKIIRIIKPYYWEVIKNKKIPMKRKVQC 300

||||||||||||||||||||||||||||||||||||||||||||||||||

33F_WciF 251 AKRVIGSFILSNSAFYNSKNDITKIIRIIKPYYWEVIKNKKIPMKRKVQC 300

33F-1_WciF 301 VLFLLSPNYYYKIKDKMLQRGRI 323

|||||||||||||||||||||||

33F_WciF 301 VLFLLSPNYYYKIKDKMLQRGRI 323

**Wzy (Identity: 99.8, Similarity: 99.8%)**

33F-1_Wzy 1 MHVRLDGLLDYIFLFSVIITCNTMYSTSQGFDGLGKWATLLLVVSVILKL 50

||||||||||||||||||||||||||||||||||||||||||||||.|||

33F_Wzy 1 MHVRLDGLLDYIFLFSVIITCNTMYSTSQGFDGLGKWATLLLVVSVFLKL 50

33F-1_Wzy 51 LISRISMKAINVIVSRSLIFILIILLIVILNGFKISETSFVYYFVLFPIF 100

||||||||||||||||||||||||||||||||||||||||||||||||||

33F_Wzy 51 LISRISMKAINVIVSRSLIFILIILLIVILNGFKISETSFVYYFVLFPIF 100

33F-1_Wzy 101 MMILQMYYDVNEIANLIRKFVRIIFLLAIGSLLFWLIGSVFHIISPTVYV 150

||||||||||||||||||||||||||||||||||||||||||||||||||

33F_Wzy 101 MMILQMYYDVNEIANLIRKFVRIIFLLAIGSLLFWLIGSVFHIISPTVYV 150

33F-1_Wzy 151 LNYWNGGGIVEGYYNLHFEAQKIEILGAILIRNTGIFAEAPMWSLVLSLA 200

||||||||||||||||||||||||||||||||||||||||||||||||||

33F_Wzy 151 LNYWNGGGIVEGYYNLHFEAQKIEILGAILIRNTGIFAEAPMWSLVLSLA 200

33F-1_Wzy 201 LIFQTLHIKKWNFTTWTLIITIMTTTSTTGVYIIGLIFLYVLFSKTSGVK 250

||||||||||||||||||||||||||||||||||||||||||||||||||

33F_Wzy 201 LIFQTLHIKKWNFTTWTLIITIMTTTSTTGVYIIGLIFLYVLFSKTSGVK 250

33F-1_Wzy 251 RYVSSLFILAIICCFSILWDNKSGTGSATIRFDDYKAGFLAWQKSPIWGL 300

||||||||||||||||||||||||||||||||||||||||||||||||||

33F_Wzy 251 RYVSSLFILAIICCFSILWDNKSGTGSATIRFDDYKAGFLAWQKSPIWGL 300

33F-1_Wzy 301 GISDGLRTIEQHMDRTVRYNLGYSNSFFVVLAQGGIMLASYYFYPVIKII 350

||||||||||||||||||||||||||||||||||||||||||||||||||

33F_Wzy 301 GISDGLRTIEQHMDRTVRYNLGYSNSFFVVLAQGGIMLASYYFYPVIKII 350

33F-1_Wzy 351 LNKFSSNDLKFSALLIIFLMITTIFIETYMFLFVISLYYSLDFGDDRDCH 400

||||||||||||||||||||||||||||||||||||||||||||||||||

33F_Wzy 351 LNKFSSNDLKFSALLIIFLMITTIFIETYMFLFVISLYYSLDFGDDRDCH 400

33F-1_Wzy 401 EKQYITN 407

|||||||

33F_Wzy 401 EKQYITN 407

**Wzx (Identity: 100%)**

33F-1_Wzx 1 MKILKNYAYNLSYQLLVIILPIITTPYVTRVFSSDDLGTYGYFSSIVTYF 50

||||||||||||||||||||||||||||||||||||||||||||||||||

33F_Wzx 1 MKILKNYAYNLSYQLLVIILPIITTPYVTRVFSSDDLGTYGYFSSIVTYF 50

33F-1_Wzx 51 TLLATLGVANYGTKEISAHRKEIGKNFWGIYSLQFGATWLSILLYLALCF 100

||||||||||||||||||||||||||||||||||||||||||||||||||

33F_Wzx 51 TLLATLGVANYGTKEISAHRKEIGKNFWGIYSLQFGATWLSILLYLALCF 100

33F-1_Wzx 101 LFTSMQNPVAYILGLSLVSKGLDISWLFQGLEDFRKITVRNITVKLVGAI 150

||||||||||||||||||||||||||||||||||||||||||||||||||

33F_Wzx 101 LFTSMQNPVAYILGLSLVSKGLDISWLFQGLEDFRKITVRNITVKLVGAI 150

33F-1_Wzx 151 SIFLFVKSANDLYLYVFLLTIFELLGQLSMWLPARQFIGKSYFDWKYAKK 200

||||||||||||||||||||||||||||||||||||||||||||||||||

33F_Wzx 151 SIFLFVKSANDLYLYVFLLTIFELLGQLSMWLPARQFIGKSYFDWKYAKK 200

33F-1_Wzx 201 HLKPVILLFLPQIAISLYVTLDRTMLGVLASTKDVGIYDQALKLVNILLT 250

||||||||||||||||||||||||||||||||||||||||||||||||||

33F_Wzx 201 HLKPVILLFLPQIAISLYVTLDRTMLGVLASTKDVGIYDQALKLVNILLT 250

33F-1_Wzx 251 LVTSLGSVMLPRVSSLLSSGDYKAVNKMHEMSFLIYNLVIFPIIAGMLIV 300

||||||||||||||||||||||||||||||||||||||||||||||||||

33F_Wzx 251 LVTSLGSVMLPRVSSLLSSGDYKAVNKMHEMSFLIYNLVIFPIIAGMLIV 300

33F-1_Wzx 301 NDDFVRFFLGQNFQDARYAIAIMTFRMFFIGWTNIMGIQILIPHNKNKEF 350

||||||||||||||||||||||||||||||||||||||||||||||||||

33F_Wzx 301 NDDFVRFFLGQNFQDARYAIAIMTFRMFFIGWTNIMGIQILIPHNKNKEF 350

33F-1_Wzx 351 MVSTTVPAILSVGLNLLLLPQFGYIGAAIVSVLTEAFVWGIQLFYTRFYL 400

||||||||||||||||||||||||||||||||||||||||||||||||||

33F_Wzx 351 MVSTTVPAILSVGLNLLLLPQFGYIGAAIVSVLTEAFVWGIQLFYTRFYL 400

33F-1_Wzx 401 KEVPIIGSMTKTALASAVMYGLLLGSKTSIHFSPTINVLIFSVLGGIIYL 450

||||||||||||||||||||||||||||||||||||||||||||||||||

33F_Wzx 401 KEVPIIGSMTKTALASAVMYGLLLGSKTSIHFSPTINVLIFSVLGGIIYL 450

33F-1_Wzx 451 FAILSLKVVDVKELKQIIRKN 471

|||||||||||||||||||||

33F_Wzx 451 FAILSLKVVDVKELKQIIRKN 471

**WciG (Identity: 85.2%, Similarity: 92.8%)**

33F-1_WciG 1 MSKFRNINLDLLKVLACVGVVLLHTTMGGFKETGAWNFLTYLYYLGTYSI 50

||||||||||||||||||||||||||||||||||||||||||||||||||

33F_WciG 1 MSKFRNINLDLLKVLACVGVVLLHTTMGGFKETGAWNFLTYLYYLGTYSI 50

33F-1_WciG 51 PLFFMVNGYLLLGKREITYSYILQKIKWLLITVSSWTFIVWLFKRDFTEN 100

||||||||||||||||||||||||||||||||||||||||||||||||||

33F_WciG 51 PLFFMVNGYLLLGKREITYSYILQKIKWLLITVSSWTFIVWLFKRDFTEN 100

33F-1_WciG 101 LIKKIIGSLIQKGYFFQFWFFGALILIYICLPVLKNFLNLKRNYLYILSV 150

||||||||||||||||||||||||||||:|||:|:.|||.||:|||.||:

33F_WciG 101 LIKKIIGSLIQKGYFFQFWFFGALILIYLCLPILRQFLNSKRSYLYSLSL 150

33F-1_WciG 151 LLVIGLIFELANIVFQMPAQTYVIQTFRLWTWFFYYLLGGFIAQFNKDII 200

|:.|||||||:||:.|||.|||||||||||||||||||||:||||..:.|

33F_WciG 151 LMTIGLIFELSNILLQMPIQTYVIQTFRLWTWFFYYLLGGYIAQFTIEEI 200

33F-1_WciG 201 RNRFKIWMKVATVLLLLISPLILFFLAKTTYHNLFAEYFYDILFVKVVSL 250

.:|||.|||:.::|||||||:||||:|||.|||||||||||.|||||.:|

33F_WciG 201 ESRFKNWMKIVSILLLLISPIILFFIAKTIYHNLFAEYFYDTLFVKVSTL 250

33F-1_WciG 251 GIFLTIFSLALNQDINKWIIFLSNQTMGVFIIHTYIMKVWEKLFGFSFVG 300

||||||..|.||::..:.|:.|||||||||||||||||||||:.||:|||

33F_WciG 251 GIFLTILMLTLNENRRESIVSLSNQTMGVFIIHTYIMKVWEKVLGFNFVG 300

33F-1_WciG 301 AYLLFAIFTLSISFIIAGMLMKIPYFNRIVKL 332

||||||:||||:||||.|||||||||||||||

33F_WciG 301 AYLLFALFTLSVSFIIVGMLMKIPYFNRIVKL 332

**Glf (Identity: 94.6%, Similarity: 96.7%)**

33F-1_Glf 1 MYDYLIVGAGLSGAIFAHEATKRGKKVKVIDKRDHIGGNIYCENVEGINV 50

|||||||||||||||||:|||||||||||||||:|||||||||||||:||

33F_Glf 1 MYDYLIVGAGLSGAIFAYEATKRGKKVKVIDKRNHIGGNIYCENVEGVNV 50

33F-1_Glf 51 HKYGAHIFHTSNKKVWDYVNQFAEFNNYINSPVANYKGSLYNLPFNMNTF 100

||||||||||||||||||||||||||||:|||||||||||||||||||||

33F_Glf 51 HKYGAHIFHTSNKKVWDYVNQFAEFNNYVNSPVANYKGSLYNLPFNMNTF 100

33F-1_Glf 101 YAMWGTKTPQEVKDKIAEQTAHMKDIEPKNLEEQAIKLIGPDVYEKLIKG 150

||||||||||||||||.||||.|||:||||||||||||||||||||||||

33F_Glf 101 YAMWGTKTPQEVKDKITEQTADMKDVEPKNLEEQAIKLIGPDVYEKLIKG 150

33F-1_Glf 151 YTEKQWGRSATELPPFIIKRLPVRLTFDNNYFNDRYQGIPIGGYNVIIEN 200

||||||||||||||||||||||||||||||||||||||||||||||||||

33F_Glf 151 YTEKQWGRSATELPPFIIKRLPVRLTFDNNYFNDRYQGIPIGGYNVIIEN 200

33F-1_Glf 201 MLKDVEVELGVDFFTHREELEASAEKVVFTGMIDQYFDYKHGELEYRSLR 250

||||||||||||||.||||||||||||||||||||||.||||||||||||

33F_Glf 201 MLKDVEVELGVDFFAHREELEASAEKVVFTGMIDQYFGYKHGELEYRSLR 250

33F-1_Glf 251 FDHEILAEENYQGNAVVNYTEREIPYTRIIEHKHFEYGTQAKTVITREYP 300

||||||.|||||||||||||||||||||||||||||||||||||||||||

33F_Glf 251 FDHEILDEENYQGNAVVNYTEREIPYTRIIEHKHFEYGTQAKTVITREYP 300

33F-1_Glf 301 ADWKRGDEPYYPINDERNNAMFAKYQEEAAQNDKVIFCGRLADYKYYDMH 350

||||||||||||||||:||||||||||||::|||||||||||||||||||

33F_Glf 301 ADWKRGDEPYYPINDEKNNAMFAKYQEEASKNDKVIFCGRLADYKYYDMH 350

33F-1_Glf 351 VVIERALEVVLSELGK--- 366

||||||||||..|.|.

33F_Glf 351 VVIERALEVVEKEFGYDKK 369

**S3 Fig. Amino acid alignment of proteins encoded in 33F and 33F-1 *cps* loci.** Identical, conserved and semi-conserved residues are denoted by the symbols ‘*’, ‘:’ and ‘.’, respectively.
