# Supplementary material for: Discovery of a Streptococcus pneumoniae serotype 33F capsular polysaccharide locus that lacks wcjE and contains a wcyO pseudogene
Source: PLoS One. 2018 Nov 5;13(11):e0206622. doi: 10.1371/journal.pone.0206622 (PMC6218050; doi:10.1371/journal.pone.0206622)
Supplement: S4 Fig — Identical nucleotides are noted by ‘|’ and differences are highlighted by ‘:’. (DOCX) [file pone.0206622.s006.docx]

| **Features [33F_cps ]:**  **wzg : [1 : 1446]**  **wzh : [1448 : 2179]**  **wzd : [2185 : 2880]**  **wze : [2890 : 3579]**  **wchA : [3594 : 4961]**  **wciB : [4948 : 5754]**  **wciC : [5758 : 6798]**  **wciD : [6795 : 7793]**  **wciE : [7795 : 8763]**  **wciF : [8783 : 9754]**  **wzy : [9755 : 10978]**  **wzx : [11009 : 12424]**  **wciG : [12426 : 13424]**  **glf : [13438 : 14547]**  **wcjE : [14534 : 15563]** | **Features [33F-1_cps]:**  **wzg : [1 : 1446]**  **wzh : [1448 : 2179]**  **wzd : [2185 : 2880]**  **wze : [2890 : 3579]**  **wchA : [3594 : 4961]**  **wciB : [4948 : 5754]**  **wciC : [5758 : 6798]**  **wciD : [6795 : 7793]**  **wciE : [7795 : 8763]**  **wciF : [8783 : 9754]**  **wzy : [9755 : 10978]**  **wzx : [11009 : 12424]**  **wciG : [12426 : 13424]**  **glf : [13438 : 14538]**  **wcyO : [14619 : 15678]** |
| --- | --- |

**33F_cps 1 ATGAGTAGACGTTTTAAAAAATCACGTTCACAGAAAGTGAAGCGAAGTGTTAATATAGTT 60**

**||||||||||||||||||||||||||||||||||||||||||||||||||||||||||||**

**33F-1_cps 1 ATGAGTAGACGTTTTAAAAAATCACGTTCACAGAAAGTGAAGCGAAGTGTTAATATAGTT 60**

**33F_cps 61 TTGCTGACTATTTATTTATTGTTAGTTTGTTTTTTATTGTTCTTAATCTTTAAGTACAAT 120**

**|||||||||||||||||||||||||||:||||||||||||||||||||||||||||||||**

**33F-1_cps 61 TTGCTGACTATTTATTTATTGTTAGTTGGTTTTTTATTGTTCTTAATCTTTAAGTACAAT 120**

**33F_cps 121 ATCCTTGCTTTTAGATATCTTAATCTAGTGGTAACTGCGTTAGTCCTACTAGTTGCCTTG 180**

**|||||||||||||||||||||||:||||||||||||||||||||||||||||||||||||**

**33F-1_cps 121 ATCCTTGCTTTTAGATATCTTAACCTAGTGGTAACTGCGTTAGTCCTACTAGTTGCCTTG 180**

**33F_cps 181 GTAGGGCTACTCTTGATTATCTATAAAAAAGCTGAAAAATTTACTATTTTTCTGTTGCTG 240**

**||||||||||||||||||||||||||||||||||||||:||||||||||||||||||:||**

**33F-1_cps 181 GTAGGGCTACTCTTGATTATCTATAAAAAAGCTGAAAAGTTTACTATTTTTCTGTTGGTG 240**

**33F_cps 241 TTCTCTATCCTTGTCAGCTCTGTGTCGCTCTTTGCAGTACAGCAGTTTGTTGGACTGACC 300**

**||||||||||||||||||||||||||||||||||||||||||||||||||||||||||||**

**33F-1_cps 241 TTCTCTATCCTTGTCAGCTCTGTGTCGCTCTTTGCAGTACAGCAGTTTGTTGGACTGACC 300**

**33F_cps 301 AATCGTTTAAATGCGACTTCTAATTACTCAGAATATTCAATCAGTGTCGCTGTTTTAGCA 360**

**||||||||||||||||||||||||||||||||||||||||||||||||||||||||||||**

**33F-1_cps 301 AATCGTTTAAATGCGACTTCTAATTACTCAGAATATTCAATCAGTGTCGCTGTTTTAGCA 360**

**33F_cps 361 GATAGTGAGATCGAAAATGTTACGCAACTGACGAGTGTGACAGCACCGACTGGGACTGAT 420**

**||||||||||||||||||||||||||||||||||||||||||||||||||||||||||||**

**33F-1_cps 361 GATAGTGAGATCGAAAATGTTACGCAACTGACGAGTGTGACAGCACCGACTGGGACTGAT 420**

**33F_cps 421 AATGAAAATATTCAAAAACTACTAGCTGATATCAAGTCAAGTCAGAATACCGATTTGACG 480**

**||||||||||||||:|||||||||||||||||||||||||||||||||||||||||||:|**

**33F-1_cps 421 AATGAAAATATTCAGAAACTACTAGCTGATATCAAGTCAAGTCAGAATACCGATTTGATG 480**

**33F_cps 481 GTCAACCAGAGTTCGTCTTACTTGGCAGCTTACAAGAGTTTGATTGCAGGGGAGACTAAG 540**

**||||||||||||||||||||||||||||||||||||||||||||||||||||||||||||**

**33F-1_cps 481 GTCAACCAGAGTTCGTCTTACTTGGCAGCTTACAAGAGTTTGATTGCAGGGGAGACTAAG 540**

**33F_cps 541 GCCATTGTCCTAAATAGTGTCTTTGAAAATATCATCGAGTCAGAGTATCCAGACTACGCA 600**

**|||||||||||||||||||||||||||||:||||||||||||||||||||||||||||||**

**33F-1_cps 541 GCCATTGTCCTAAATAGTGTCTTTGAAAACATCATCGAGTCAGAGTATCCAGACTACGCA 600**

**33F_cps 601 TCGAAGATAAAAAAGATTTATACCAAGGGATTCACTAAAAAAGTAGAAGCTCCTAAGACG 660**

**||||||||||||||||||||||||||||||||||||||||||||||||||||||||||||**

**33F-1_cps 601 TCGAAGATAAAAAAGATTTATACCAAGGGATTCACTAAAAAAGTAGAAGCTCCTAAGACG 660**

**33F_cps 661 TCTAAGAATCAGTCTTTCAATATCTATGTTAGTGGAATTGACACCTATGGCCCTATTAGT 720**

**||||||||||||||||||||||||||||||||||||||||||||||||||:|||||||||**

**33F-1_cps 661 TCTAAGAATCAGTCTTTCAATATCTATGTTAGTGGAATTGACACCTATGGTCCTATTAGT 720**

**33F_cps 721 TCAGTGTCGCGATCAGATGTCAATATCCTGATGACTGTCAATCGAGATACCAAGAAAATC 780**

**||:|||||||||||||||||||||||||||||||||||||||||||||||||||||||||**

**33F-1_cps 721 TCGGTGTCGCGATCAGATGTCAATATCCTGATGACTGTCAATCGAGATACCAAGAAAATC 780**

**33F_cps 781 CTCTTGACCACAACGCCACGTGATGCCTATGTACCAATCGCAGATGGTGGAAATAATCAA 840**

**||||||||||||||||||||||||||||||||||||||||||||||||||||||||||||**

**33F-1_cps 781 CTCTTGACCACAACGCCACGTGATGCCTATGTACCAATCGCAGATGGTGGAAATAATCAA 840**

**33F_cps 841 AAAGATAAATTAACCCATGCGGGCATTTATGGAGTTGATTCGTCCATTCACACCTTAGAA 900**

**||||||||||||||||||||:|||||||||||||||||||||||||||||||||||||||**

**33F-1_cps 841 AAAGATAAATTAACCCATGCAGGCATTTATGGAGTTGATTCGTCCATTCACACCTTAGAA 900**

**33F_cps 901 AATCTCTATGGAGTGGATATCAATTACTATGTGCGATTGAACTTCACTTCGTTTTTGAAA 960**

**||||||||||||||||||||||||||||||||||||||||||||||||||:||:||||||**

**33F-1_cps 901 AATCTCTATGGAGTGGATATCAATTACTATGTGCGATTGAACTTCACTTCTTTCTTGAAA 960**

**33F_cps 961 TTGATTGATTTGTTGGGTGGAATTGATGTTTATAATGATCAAGAATTTACTGCCCATACG 1020**

**:|||||||:||:|||||:||::|:||||||:|||||||||||||:|||:|:||:|:::::**

**33F-1_cps 961 ATGATTGACTTATTGGGAGGGGTAGATGTTCATAATGATCAAGAGTTTTCAGCTCTACAT 1020**

**33F_cps 1021 AATGGAAAGTATTACCCTGCAGGCAATGTTCATCTTGATTCAGAACAGGCTCTCGGTTTT 1080**

**::::::::::|||:|||:|:|||:|||||:|||||:||:||:||:||||||||:||||||**

**33F-1_cps 1021 GGGAAGTTCCATTTCCCAGTAGGGAATGTCCATCTAGACTCTGAGCAGGCTCTAGGTTTT 1080**

**33F_cps 1081 GTTCGTGAGCGCTACTCACTAGCAGATGGCGATCGTGACCGCGGGCGCAATCAACAAAAG 1140**

**||:|||||:||||||||||||||:|||||:||:|||||:|||||||||||||||||||||**

**33F-1_cps 1081 GTACGTGAACGCTACTCACTAGCCGATGGAGACCGTGATCGCGGGCGCAATCAACAAAAG 1140**

**33F_cps 1141 GTGATTGTGGCTATCCTTCAAAAATTAACGTCAACCGAAGCACTGAAAAATTATAGCACG 1200**

**||||||||||||||||||||||||||||||||||||||||||||||||||||||||:|||**

**33F-1_cps 1141 GTGATTGTGGCTATCCTTCAAAAATTAACGTCAACCGAAGCACTGAAAAATTATAGTACG 1200**

**33F_cps 1201 ATCATTAATAGCTTGCAAGATTCTATCCAAACAAATATGCCACTTGAGACTATGATAAAT 1260**

**||||||||||||||||||||||||||||||||||||||||||||||||||||||||||||**

**33F-1_cps 1201 ATCATTAATAGCTTGCAAGATTCTATCCAAACAAATATGCCACTTGAGACTATGATAAAT 1260**

**33F_cps 1261 TTGGTCAATGCTCAGTTAGAAAGTGGAGGGAATTATAAAGTAAATTCTCAAGATTTAAAA 1320**

**||||||||||||||||||||||||||||||||||||||||||||||||||||||||||||**

**33F-1_cps 1261 TTGGTCAATGCTCAGTTAGAAAGTGGAGGGAATTATAAAGTAAATTCTCAAGATTTAAAA 1320**

**33F_cps 1321 GGTACAGGTCGGACGGATCTTCCTTCTTATGCAATGCCAGACAGTAACCTCTATGTGATG 1380**

**||:||||||||||:||||||||||||||||||||||||||||||||||||||||||||||**

**33F-1_cps 1321 GGGACAGGTCGGATGGATCTTCCTTCTTATGCAATGCCAGACAGTAACCTCTATGTGATG 1380**

**33F_cps 1381 GAAATAGATGATAGTAGTTTAGCTGTAGTTAAAGCAGCTATACAGGATGTGATGGAGGGT 1440**

**||||||||||||||||||||||||||||||||||||||||||||||||||||||||||||**

**33F-1_cps 1381 GAAATAGATGATAGTAGTTTAGCTGTAGTTAAAGCAGCTATACAGGATGTGATGGAGGGT 1440**

**33F_cps 1441 AGATGAAATGATAGACATCCATTCGCATATCGTTTTTGATGTAGATGACGGTCCCAAGTC 1500**

**|||||||||||||||||||||||||||:|||||:||||||||||||||:|||||||||||**

**33F-1_cps 1441 AGATGAAATGATAGACATCCATTCGCACATCGTCTTTGATGTAGATGATGGTCCCAAGTC 1500**

**33F_cps 1501 AAGAGAGGAAAGCAAGGCTCTCTTGACAGAATCCTACAGGCAGGGGGTGAGAACCATTGT 1560**

**|||||||||||||||||||||||||:|||||||||||||:|||||||||:||||||||||**

**33F-1_cps 1501 AAGAGAGGAAAGCAAGGCTCTCTTGGCAGAATCCTACAGACAGGGGGTGCGAACCATTGT 1560**

**33F_cps 1561 CTCTACCTCTCACCGTCGCAAGGGCATGTTTGAAACTCCGGAAGAGAAGATAGCAGAAAA 1620**

**||||||||||||||||||||||||||||||||||||||||||||||||||||||||||||**

**33F-1_cps 1561 CTCTACCTCTCACCGTCGCAAGGGCATGTTTGAAACTCCGGAAGAGAAGATAGCAGAAAA 1620**

**33F_cps 1621 CTTTCTTCAGGTTCGGGAAATAGCTAAGGAAGTGGCGAGTGACTTGGTCATTGCTTATGG 1680**

**||||||||||||||||||||||||||||||||||||||||||||||:||||||||||:||**

**33F-1_cps 1621 CTTTCTTCAGGTTCGGGAAATAGCTAAGGAAGTGGCGAGTGACTTGATCATTGCTTACGG 1680**

**33F_cps 1681 GGCTGAAATTTACTACACACCAGATGTTCTGGATAAGCTGGAAAAAAAGCGGATTCCGAC 1740**

**||||||||||||||||||||||||||||||||||||||||||||||||||||||||||||**

**33F-1_cps 1681 GGCTGAAATTTACTACACACCAGATGTTCTGGATAAGCTGGAAAAAAAGCGGATTCCGAC 1740**

**33F_cps 1741 CCTCAATGATAGTCGTTATGCCTTGATAGAGTTCAGTATGAACACTCCTTATCGCGATAT 1800**

**|||||||||||||||||||||||||||||||||:||||||||||||||||||||||||||**

**33F-1_cps 1741 CCTCAATGATAGTCGTTATGCCTTGATAGAGTTTAGTATGAACACTCCTTATCGCGATAT 1800**

**33F_cps 1801 TCATAGCGCCTTGAGCAAGATCTTGATGTTGGGAATTACTCCAGTCATTGCCCACATTGA 1860**

**||||||||||||||||||||||||||||||||||||||||||||||||||||||||||||**

**33F-1_cps 1801 TCATAGCGCCTTGAGCAAGATCTTGATGTTGGGAATTACTCCAGTCATTGCCCACATTGA 1860**

**33F_cps 1861 GCGCTATGATGCTCTTGAAAATAATGAAAAACGCGTTCGAGAACTGATTGATAGGGGCTG 1920**

**|||||||||||||||||||||||||||||||||||||||||||||||||||||:||||||**

**33F-1_cps 1861 GCGCTATGATGCTCTTGAAAATAATGAAAAACGCGTTCGAGAACTGATTGATATGGGCTG 1920**

**33F_cps 1921 TTACACGCAAGTAAATAGTTCACATGTCCTCAAACCCAAACTTTTTGGCGAACGTTATAA 1980**

**||||||||||||||||||||||||||||||||||:|||||||||||||||||||||||||**

**33F-1_cps 1921 TTACACGCAAGTAAATAGTTCACATGTCCTCAAATCCAAACTTTTTGGCGAACGTTATAA 1980**

**33F_cps 1981 ATTCATGAAAAAAAGAGCTCAGTATTTTTTAGAGCAGGATTTGGTTCATATCATTGCAAG 2040**

**|||||||||||||||||||||||||||||||||||||||||||||:|||:||||||||||**

**33F-1_cps 1981 ATTCATGAAAAAAAGAGCTCAGTATTTTTTAGAGCAGGATTTGGTCCATGTCATTGCAAG 2040**

**33F_cps 2041 TGATATGCACAATCTAGACGGTAGACCTCCTCATATGGCAGAAGCATATGACCTTGTTAC 2100**

**|||||||||||||||||||||:||||||||||||||||||||||||||||||||||||||**

**33F-1_cps 2041 TGATATGCACAATCTAGACGGCAGACCTCCTCATATGGCAGAAGCATATGACCTTGTTAC 2100**

**33F_cps 2101 CCAAAAATACGGAGAAGCGAAGGCTCAGGAACTTTTTATAGACAATCCTCGAAAAATTGT 2160**

**||||||||||||||||||||||||||||||||||||||||||||||||||||||||||:|**

**33F-1_cps 2101 CCAAAAATACGGAGAAGCGAAGGCTCAGGAACTTTTTATAGACAATCCTCGAAAAATTAT 2160**

**33F_cps 2161 AATGGATCAACTAATTTAGGAGAAATGATGAAAGAACAAAACACGATAGAAATCGATGTA 2220**

**|||||||||||||||||||||||||||||||||||||||||||:||||||||||||||||**

**33F-1_cps 2161 AATGGATCAACTAATTTAGGAGAAATGATGAAAGAACAAAACATGATAGAAATCGATGTA 2220**

**33F_cps 2221 TTTCAATTATTTAAAACCTTGTGGAAACGCAAGCTAATGATTTTAATAGTGGCACTTGTG 2280**

**||||||||||||||||||||||||||||||||||||||:|||||||||||||||||||||**

**33F-1_cps 2221 TTTCAATTATTTAAAACCTTGTGGAAACGCAAGCTAATAATTTTAATAGTGGCACTTGTG 2280**

**33F_cps 2281 ACAGGTGCGGGGGCTTTTGCATATAGCACTTTTATTGTTAAGCCAGAATATACGAGTACC 2340**

**||||||||||||||||||||||||||||||||||||||||||||||||||||||||||||**

**33F-1_cps 2281 ACAGGTGCGGGGGCTTTTGCATATAGCACTTTTATTGTTAAGCCAGAATATACGAGTACC 2340**

**33F_cps 2341 ACGCGAATTTACGTAGTGAATCGCAATCAAGGAGACAAGCCGGGGCTGACAAATCAGGAT 2400**

**||||||||||||||||||||||||:|||||||||||||||||||||||||||||||||||**

**33F-1_cps 2341 ACGCGAATTTACGTAGTGAATCGCGATCAAGGAGACAAGCCGGGGCTGACAAATCAGGAT 2400**

**33F_cps 2401 TTGCAGGCAGGATCTTATCTGGTAAAAGACTACCGTGAGATTATCCTTTCGCAGGATGCA 2460**

**||||||||||||||||||||||||||||||||||||||||||||||||||||||||||:|**

**33F-1_cps 2401 TTGCAGGCAGGATCTTATCTGGTAAAAGACTACCGTGAGATTATCCTTTCGCAGGATGTA 2460**

**33F_cps 2461 TTGGAAAAAGTAGCGACAAATTTGAAGTTGGATATGCCAGCAAAAACGTTAGCCAGCAAA 2520**

**||||||||||||||||||||||||||||||||||||||||||||||||||||||||||||**

**33F-1_cps 2461 TTGGAAAAAGTAGCGACAAATTTGAAGTTGGATATGCCAGCAAAAACGTTAGCCAGCAAA 2520**

**33F_cps 2521 GTTCAAGTGACTGTACCAGCTGACACTCGTATCGTCTCAATCTCTGTCAAGGATAAACAG 2580**

**||||||||||||||||||||||||||||||||||||||||||||||||||||||||||||**

**33F-1_cps 2521 GTTCAAGTGACTGTACCAGCTGACACTCGTATCGTCTCAATCTCTGTCAAGGATAAACAG 2580**

**33F_cps 2581 CCAGAGGAAGCCAGTCGTATCGCTAATTCTCTACGAGAAGTTGCTGCAGAAAAGATCGTC 2640**

**||||||||||||||||||||||||||||||||||||||||||||||||||||||||||||**

**33F-1_cps 2581 CCAGAGGAAGCCAGTCGTATCGCTAATTCTCTACGAGAAGTTGCTGCAGAAAAGATCGTC 2640**

**33F_cps 2641 GCTGTAACGCGAGTATCTGATGTAACGACACTTGAAGAAGCGCGACCAGCTACGACTCCC 2700**

**|||||||||||||||||||||||||||||||||||||||||||||||||||||||||||:**

**33F-1_cps 2641 GCTGTAACGCGAGTATCTGATGTAACGACACTTGAAGAAGCGCGACCAGCTACGACTCCT 2700**

**33F_cps 2701 TCTTCTCCAAATGTTCGACGCAATTCCTTGTTTGGTTTTCTTGGAGGAGCAGTCGTAACA 2760**

**||||||||||||||||||||||||||||||||||||||||||||||||||||||||||||**

**33F-1_cps 2701 TCTTCTCCAAATGTTCGACGCAATTCCTTGTTTGGTTTTCTTGGAGGAGCAGTCGTAACA 2760**

**33F_cps 2761 GTAATTGCTGTTCTTTTGATTGAGTTGCTGGACACCCGTGTGAAACGTCCTGAAGATGTT 2820**

**||||||||||||||||||||||||:|||||||||||||||||||||||||||||||||||**

**33F-1_cps 2761 GTAATTGCTGTTCTTTTGATTGAGGTGCTGGACACCCGTGTGAAACGTCCTGAAGATGTT 2820**

**33F_cps 2821 GAAGATGTACTGAAAATTCCACTTTTAGGGCTCGTTCCAGATTTTGACAAAATAAAATAG 2880**

**||||||||||||||||||||||||||||||||||||||||||||||||||||||||||||**

**33F-1_cps 2821 GAAGATGTACTGAAAATTCCACTTTTAGGGCTCGTTCCAGATTTTGACAAAATAAAATAG 2880**

**33F_cps 2881 GAGGAAGTTATGCCAACATTAGAAATCTCACAGGCAAAATTGGATTCTGTAAAAAAGGCA 2940**

**||||||||||||||||||||||||||||||||||||||||||||||||||||||||||||**

**33F-1_cps 2881 GAGGAAGTTATGCCAACATTAGAAATCTCACAGGCAAAATTGGATTCTGTAAAAAAGGCA 2940**

**33F_cps 2941 GAGGAATATTATAACGCTTTGTGCACGAACCTACAGTTAAGTGGAGATGGTTTGAAAGTA 3000**

**||||||||||||||||||||||||||||||||||||||||||||||||||||||||||||**

**33F-1_cps 2941 GAGGAATATTATAACGCTTTGTGCACGAACCTACAGTTAAGTGGAGATGGTTTGAAAGTA 3000**

**33F_cps 3001 TTTTCTATCACTTCTGTGAAAATAGGAGAAGGAAAATCAACGACTTCCGCCAATATCGCT 3060**

**||:|||||||||||||||||||||||||||||||||||||||||||||||||||||||||**

**33F-1_cps 3001 TTGTCTATCACTTCTGTGAAAATAGGAGAAGGAAAATCAACGACTTCCGCCAATATCGCT 3060**

**33F_cps 3061 TGGGCTTTTGCGCGTGCAGGTTACAAAACGCTGCTGATTGATGGAGATATTCGCAATTCT 3120**

**||||||||||||||||||||||||||||||||||||||||||||||||||||||||||||**

**33F-1_cps 3061 TGGGCTTTTGCGCGTGCAGGTTACAAAACGCTGCTGATTGATGGAGATATTCGCAATTCT 3120**

**33F_cps 3121 GTTATGTTAGGTGTCTTTAAAGCAAGGAATAAGATTACAGGCCTGACAGAATTTTTATCA 3180**

**||||||||||||||||||||||||||||||||||||||||||||||||||||||||||||**

**33F-1_cps 3121 GTTATGTTAGGTGTCTTTAAAGCAAGGAATAAGATTACAGGCCTGACAGAATTTTTATCA 3180**

**33F_cps 3181 GGAACTACAGACTTATCACAAGGGCTTTGTGATACCAATATCGAAAATCTCTTTGTAATT 3240**

**||||||||||||:|||||||||||||||||||||||||||||||||||||||||||||||**

**33F-1_cps 3181 GGAACTACAGACCTATCACAAGGGCTTTGTGATACCAATATCGAAAATCTCTTTGTAATT 3240**

**33F_cps 3241 CAGGCTGGCTCTGTGTCACCGAATCCGACAGCTCTTCTTCAAAGTAAGAATTTCACTACA 3300**

**||||||||||||||||||||||||||||||||||||||||||||||||||||||||||||**

**33F-1_cps 3241 CAGGCTGGCTCTGTGTCACCGAATCCGACAGCTCTTCTTCAAAGTAAGAATTTCACTACA 3300**

**33F_cps 3301 ATGCTTGAAACCTTGCGTAAATATTTTGACTACATCATTGTAGATACTGCTCCTGTTGGT 3360**

**||||||||||||||||||||||||||||||||||||||||||||||||||||||||||||**

**33F-1_cps 3301 ATGCTTGAAACCTTGCGTAAATATTTTGACTACATCATTGTAGATACTGCTCCTGTTGGT 3360**

**33F_cps 3361 GTCGTGATTGATGCGGCTATTATTACGCGAAACTGCGATGCTTCTATTTTAGTGACGGAG 3420**

**||||||||||||||||||||||||||||||||||||||||||||||||||||||||||||**

**33F-1_cps 3361 GTCGTGATTGATGCGGCTATTATTACGCGAAACTGCGATGCTTCTATTTTAGTGACGGAG 3420**

**33F_cps 3421 GCAGGTGAAATAAATCGTCGGGATATTCAAAAAGCAAAAGAACAGTTGGAACACACAGGA 3480**

**|||||||||||||||||:|||||||||||||||||||||||||||||||||||||||||:**

**33F-1_cps 3421 GCAGGTGAAATAAATCGACGGGATATTCAAAAAGCAAAAGAACAGTTGGAACACACAGGG 3480**

**33F_cps 3481 AAGCCGTTTTTGGGAATTGTGTTGAATAAATTCGATACTTCAGTAGACAAATACGGTTCT 3540**

**|||||||||||||||||||||||||||||||||||||||||||||:||||||||||||||**

**33F-1_cps 3481 AAGCCGTTTTTGGGAATTGTGTTGAATAAATTCGATACTTCAGTAAACAAATACGGTTCT 3540**

**33F_cps 3541 TATGGAAATTATGGAGATTACGGGAAAAATAAAAAATAGGTCGGGGGATAGAGATGAATG 3600**

**||||||||||||||||||||||||||||||||||||||||||||||||||||||||||||**

**33F-1_cps 3541 TATGGAAATTATGGAGATTACGGGAAAAATAAAAAATAGGTCGGGGGATAGAGATGAATG 3600**

**33F_cps 3601 GAAAAATAGTAAAGCCTTCATTGGCCATAATCCAGAGTTTTCTTGTTATTTTATTGACTT 3660**

**||||||||:|||:|:|||||||||:|||||||||||||||||||||||||||||||||||**

**33F-1_cps 3601 GAAAAATATTAAGGTCTTCATTGGTCATAATCCAGAGTTTTCTTGTTATTTTATTGACTT 3660**

**33F_cps 3661 ATCTACTTAGTGCTGTGAGAGAAGCGGAGATTGTTTCAACAACAGCTATTGCACTTTATA 3720**

**|||||||||||:||||||||||||||||||||||||||||||||||||||||||||||||**

**33F-1_cps 3661 ATCTACTTAGTACTGTGAGAGAAGCGGAGATTGTTTCAACAACAGCTATTGCACTTTATA 3720**

**33F_cps 3721 TCCTCCATTATTTTGTCTTTTATATCAGTGTTTATGGGCAGGATTTCTTTAAAAGGGGAT 3780**

**||||||||||||||||||||||||||||||:||||||:||||||||||||||||||||||**

**33F-1_cps 3721 TCCTCCATTATTTTGTCTTTTATATCAGTGATTATGGACAGGATTTCTTTAAAAGGGGAT 3780**

**33F_cps 3781 ATTTGATTGAACTTGTCCAGACATTGAAATATATCCTATTCTTTGCACTAGCGATTAGTA 3840**

**||||||||||||||:|||||||||||||||||||||||||||||||||||||||||||||**

**33F-1_cps 3781 ATTTGATTGAACTTCTCCAGACATTGAAATATATCCTATTCTTTGCACTAGCGATTAGTA 3840**

**33F_cps 3841 TTTCTAATTTTTTCTTAGAGGATCGATTTAGTATTTCCAGACGAGGCATGATTTACTTCC 3900**

**||||||||||||||||||||||||||||||||||||||||||||||||||||||||||||**

**33F-1_cps 3841 TTTCTAATTTTTTCTTAGAGGATCGATTTAGTATTTCCAGACGAGGCATGATTTACTTCC 3900**

**33F_cps 3901 TCACATTACATGCTCTCTTAGTCTATGTGCTAAACCTATTTATCAAGTGGTATTGGAAGC 3960**

**||||||||||||||||||||||||||||||||||||||||||||||||||||||||||||**

**33F-1_cps 3901 TCACATTACATGCTCTCTTAGTCTATGTGCTAAACCTATTTATCAAGTGGTATTGGAAGC 3960**

**33F_cps 3961 GGACTTATCCCAACTTTAAAGGAAGTAAGAAGATCCTCTTGCTTACAGCAACTTTTCGTG 4020**

**||:|||||||||||||||||||||||||||||||:|||:|:|||||||||||||:|||||**

**33F-1_cps 3961 GGGCTTATCCCAACTTTAAAGGAAGTAAGAAGATTCTCCTACTTACAGCAACTTCTCGTG 4020**

**33F_cps 4021 TCGAAAAGGTACTGGATAGATTAATAGAATCAAATGAGGTTGTTGGGGAGTTGGTAGCCG 4080**

**|||||||||||||||||||||||||||||||||||||||||||||||:||||||||||||**

**33F-1_cps 4021 TCGAAAAGGTACTGGATAGATTAATAGAATCAAATGAGGTTGTTGGGAAGTTGGTAGCCG 4080**

**33F_cps 4081 TCAGTGTCTTAGATAAACCAGATTTTCAGCATGATTGTTTAAAGGTAGTAGCAGAGGGGG 4140**

**||||||||||||||||||||||||||||||||||||||||||||||||||||||||||||**

**33F-1_cps 4081 TCAGTGTCTTAGATAAACCAGATTTTCAGCATGATTGTTTAAAGGTAGTAGCAGAGGGGG 4140**

**33F_cps 4141 AGATAGTAAACTTTGCGACTCATGAGGTGGTCGATGAAGTCTTTATCAATCTTCCGAGTG 4200**

**|||||||||||||||||||||||||||||||:|||||||||||||||||||||||:||||**

**33F-1_cps 4141 AGATAGTAAACTTTGCGACTCATGAGGTGGTAGATGAAGTCTTTATCAATCTTCCAAGTG 4200**

**33F_cps 4201 AAAAATACAATATTGGAGAGCTTGTCTCTCAGTTTGAAACGATGGGAATTGATGTAACAG 4260**

**||||||||||||||||||||||:|||||||||||||||||||||||||||||||||||||**

**33F-1_cps 4201 AAAAATACAATATTGGAGAGCTGGTCTCTCAGTTTGAAACGATGGGAATTGATGTAACAG 4260**

**33F_cps 4261 TTAATCTAAATGCTTTTGATCGTAGTTTGGCACGTAACAAGCAAATTCGTGAGATGGCAG 4320**

**||||||||||||||||||||||||||||||||||||||||||||||||||||||||||||**

**33F-1_cps 4261 TTAATCTAAATGCTTTTGATCGTAGTTTGGCACGTAACAAGCAAATTCGTGAGATGGCAG 4320**

**33F_cps 4321 GATTAAACGTTGTGACTTTTTCTACAACATTTTATAAGACTAGTCATGTAATTGCTAAGC 4380**

**||||||||||||||||||||||||||:||||||||||||||||||||||||||||||||:**

**33F-1_cps 4321 GATTAAACGTTGTGACTTTTTCTACAGCATTTTATAAGACTAGTCATGTAATTGCTAAGA 4380**

**33F_cps 4381 GGATTATTGATATCATGGGTGCATTGGTCGGGTTGATATTATGTGGTTTAGTCAGTATTG 4440**

**||||||||||||||:|||||||||||||:|||:|||||:|||||||||||||||||||||**

**33F-1_cps 4381 GGATTATTGATATCGTGGGTGCATTGGTAGGGCTGATACTATGTGGTTTAGTCAGTATTG 4440**

**33F_cps 4441 TACTGGTTCCTTTGATTCGAAAGGATGGGGGCTCTGCTATTTTTGCTCAGACGCGTATAG 4500**

**||:|||||||||||||||||||||||||||||||||||||||||||||||||||||||||**

**33F-1_cps 4441 TATTGGTTCCTTTGATTCGAAAGGATGGGGGCTCTGCTATTTTTGCTCAGACGCGTATAG 4500**

**33F_cps 4501 GAAAAAATGGTCGTCAGTTCACCTTTTATAAGTTTCGCTCTATGTGTGTAGATGCCGAGG 4560**

**||||||||||||||||||||||:|||||||||||||||||||||||||||||||||||||**

**33F-1_cps 4501 GAAAAAATGGTCGTCAGTTCACTTTTTATAAGTTTCGCTCTATGTGTGTAGATGCCGAGG 4560**

**33F_cps 4561 CGAAAAAAAGAGAACTCATGGAACAAAATACCATGCAGGGTGGAATGTTTAAGGTGGACG 4620**

**||||||||||||||||||||||||||||||||||||||||||||||||||||||||||||**

**33F-1_cps 4561 CGAAAAAAAGAGAACTCATGGAACAAAATACCATGCAGGGTGGAATGTTTAAGGTGGACG 4620**

**33F_cps 4621 ATGATCCTCGTATCACGAAAATTGGTCGTTTTATACGGAAGACTAGCTTGGACGAGCTAC 4680**

**||||||||||||||||||||||||||||||||||||||||||||||||||||||||||||**

**33F-1_cps 4621 ATGATCCTCGTATCACGAAAATTGGTCGTTTTATACGGAAGACTAGCTTGGACGAGCTAC 4680**

**33F_cps 4681 CACAGTTTTATAATGTTCTAAAGGGAGATATGAGTTTGGTTGGCACACGGCCACCAACAG 4740**

**||||||||||||||||||||||||||||||||||||||||||||||||||||||||||||**

**33F-1_cps 4681 CACAGTTTTATAATGTTCTAAAGGGAGATATGAGTTTGGTTGGCACACGGCCACCAACAG 4740**

**33F_cps 4741 TGGATGAGTATGAACACTATACCCCAGAACAAAAACGTCGGCTAAGTTTTAAACCTGGTG 4800**

**||||:||||||||||||||||||||||||||||||||||||||||||||||||||||||:**

**33F-1_cps 4741 TGGACGAGTATGAACACTATACCCCAGAACAAAAACGTCGGCTAAGTTTTAAACCTGGTA 4800**

**33F_cps 4801 TAACAGGCTTATGGCAGGTCAGTGGACGAAGTGAAATCAAAAATTTCGATGAAGTTGTCA 4860**

**||||||||||||||||||||||||||||||||||||||||||||||||||||||||||||**

**33F-1_cps 4801 TAACAGGCTTATGGCAGGTCAGTGGACGAAGTGAAATCAAAAATTTCGATGAAGTTGTCA 4860**

**33F_cps 4861 AATTAGATGTGGCTTATATAGATGATTGGACAATCTGGAAAGATATTGAAATTTTATTGA 4920**

**||||||||||||||||||||||||||||||||||||||||||||||||||||||||||||**

**33F-1_cps 4861 AATTAGATGTGGCTTATATAGATGATTGGACAATCTGGAAAGATATTGAAATTTTATTGA 4920**

**33F_cps 4921 AGACAGTTAAGGTAGTATTGATGAAGGATGGAGCGAAGTAGATTGATAGATGTAAAAATC 4980**

**||||||||||||||||||||||||||||||||||||||||||||||||||||||||||||**

**33F-1_cps 4921 AGACAGTTAAGGTAGTATTGATGAAGGATGGAGCGAAGTAGATTGATAGATGTAAAAATC 4980**

**33F_cps 4981 ATTGTGGCAACGCATAAAGAGGTTAAAATGCCTCAAGACAATAGTCTTTACCTTCCAATA 5040**

**||||||||||||||||||||||||||||||||||||||||||||||||||||||||||||**

**33F-1_cps 4981 ATTGTGGCAACGCATAAAGAGGTTAAAATGCCTCAAGACAATAGTCTTTACCTTCCAATA 5040**

**33F_cps 5041 CATGTTGGGAGAGACGGTAAATCAGATATTGGTTTTATCGGTGATAATACTGGCGATAAT 5100**

**||||||||||||||||||||||||||||||||||||||||||||||||||||||||||||**

**33F-1_cps 5041 CATGTTGGGAGAGACGGTAAATCAGATATTGGTTTTATCGGTGATAATACTGGCGATAAT 5100**

**33F_cps 5101 ATATCCTCTCTAAATCCATATTATTGTGAGTTAACGGGACTTTATTGGGCATGGAAGAAT 5160**

**||||||||||||||||||||||||||||||||||||||||||||||||||||||||||||**

**33F-1_cps 5101 ATATCCTCTCTAAATCCATATTATTGTGAGTTAACGGGACTTTATTGGGCATGGAAGAAT 5160**

**33F_cps 5161 CTTGATTATAATTACTTAGGTTTAGTTCATTACCGTCGTTATTTTACAAATAAATCTCAA 5220**

**||||||||||||||||||||||||||||||||||||||||||||||||||||||||||||**

**33F-1_cps 5161 CTTGATTATAATTACTTAGGTTTAGTTCATTACCGTCGTTATTTTACAAATAAATCTCAA 5220**

**33F_cps 5221 GGGTATAATGAAAATGTCAATATGGATGACCTCATTTTGTCTCGAGCTAATGTTGAAATA 5280**

**||||||||||||||||||||||||||||||:|||||||||||||||||||||||||||||**

**33F-1_cps 5221 GGGTATAATGAAAATGTCAATATGGATGACGTCATTTTGTCTCGAGCTAATGTTGAAATA 5280**

**33F_cps 5281 TTATTAGAGAAATCTGACATAATAGTTCCAAAGAAGCGAAAGTATTATATTGAAACTCTT 5340**

**||||||||||||||||||||||||||||||||||||||||||||||||||||||||||||**

**33F-1_cps 5281 TTATTAGAGAAATCTGACATAATAGTTCCAAAGAAGCGAAAGTATTATATTGAAACTCTT 5340**

**33F_cps 5341 TATTCACATTATGCCCATACCCTTAACGGAGAACATCTGGATCTTGCTAGGAAAATTATT 5400**

**||||||||||||||||||||||||||||||||||||||||||||||||||||||||||||**

**33F-1_cps 5341 TATTCACATTATGCCCATACCCTTAACGGAGAACATCTGGATCTTGCTAGGAAAATTATT 5400**

**33F_cps 5401 GAGCAAAATAGTTCAGAGTATCTTTCATCCTTTGATAAAGTGATGAAACAAAGAAGCGGT 5460**

**||||||||||||||||||||||||||||||||||||||||||||||||||||||||||||**

**33F-1_cps 5401 GAGCAAAATAGTTCAGAGTATCTTTCATCCTTTGATAAAGTGATGAAACAAAGAAGCGGT 5460**

**33F_cps 5461 TATATGTTCAATATGTTTATCATGAAAAAAGAACTATTAGATGATTATTTACCGTGGCTT 5520**

**||||||||||||||||||||||||||||||||||||||||||||||||||||||||||||**

**33F-1_cps 5461 TATATGTTCAATATGTTTATCATGAAAAAAGAACTATTAGATGATTATTTACCGTGGCTT 5520**

**33F_cps 5521 TTTTCTATTCTGGATACTATGTACGAACAGATGGACTTGACTGACTATACTTTATTTGAG 5580**

**||||||||||||||||||||||||||||||||||||||||||||||||||||||||||||**

**33F-1_cps 5521 TTTTCTATTCTGGATACTATGTACGAACAGATGGACTTGACTGACTATACTTTATTTGAG 5580**

**33F_cps 5581 TCACGTTTATTCGGGCGAGTTAGTGAGTTGTTGTTTAATGTTTGGTTATGTCAAAAAGGA 5640**

**|||||||||||||||||||||||||||||||||||||||||||||||||||:||||||||**

**33F-1_cps 5581 TCACGTTTATTCGGGCGAGTTAGTGAGTTGTTGTTTAATGTTTGGTTATGTAAAAAAGGA 5640**

**33F_cps 5641 ATAACGCCTAAAGAGGTACCATTTATGTACATGGAGAGAGTGGATTTGTTTGAAAAAGGA 5700**

**||||||||||||||||||||||||||||||||||||||||||||||||||||||||||||**

**33F-1_cps 5641 ATAACGCCTAAAGAGGTACCATTTATGTACATGGAGAGAGTGGATTTGTTTGAAAAAGGA 5700**

**33F_cps 5701 AAATCTTTTTTAATGGCTAAATTTTTTGGAAAGAAGTATGGACAGAGTTTCTAGGATATG 5760**

**||||||||||||||||||||||||||||||||||||||||||||||||||||||||||||**

**33F-1_cps 5701 AAATCTTTTTTAATGGCTAAATTTTTTGGAAAGAAGTATGGACAGAGTTTCTAGGATATG 5760**

**33F_cps 5761 AAAGTAACTATTATTGGACAAATAAAAAATAAGAGAACAGGTCTTGGAAAGGCGATTAAT 5820**

**||||||||||||||||||||||||||||||||||||||||||||||||||||||||||||**

**33F-1_cps 5761 AAAGTAACTATTATTGGACAAATAAAAAATAAGAGAACAGGTCTTGGAAAGGCGATTAAT 5820**

**33F_cps 5821 GATTTTAGGGATTATTGTTGCAATCGTGCTACAAGAGTAACGGAAATAGATATAACAAAT 5880**

**||||||||||||||||||||||||||||||||||||||||||||||||||||||||||||**

**33F-1_cps 5821 GATTTTAGGGATTATTGTTGCAATCGTGCTACAAGAGTAACGGAAATAGATATAACAAAT 5880**

**33F_cps 5881 AATTTTAATTTTTTAAGCTCTCTTTTTCAAATTTTAATATCTGATACAGATGTTTATTAT 5940**

**||||||||||||||||||||||||||||||||||||||||||||||||||||||||||||**

**33F-1_cps 5881 AATTTTAATTTTTTAAGCTCTCTTTTTCAAATTTTAATATCTGATACAGATGTTTATTAT 5940**

**33F_cps 5941 TTTACTCCAGCTGGTTCAGTAGCTGGTAATATTAGGGATTCACTGTTTTTGTTCTTTATG 6000**

**||||||||||||||||||||||||||||||||||||||||||||||||||||||||||||**

**33F-1_cps 5941 TTTACTCCAGCTGGTTCAGTAGCTGGTAATATTAGGGATTCACTGTTTTTGTTCTTTATG 6000**

**33F_cps 6001 ATAATGAAAAGGAAAAAAATAGTGACCCATTTTCATAATAGTGCTTTTGGAAATGTGATG 6060**

**||||||||||||||||||||||||||||||||||||||||||||||||||||||||||||**

**33F-1_cps 6001 ATAATGAAAAGGAAAAAAATAGTGACCCATTTTCATAATAGTGCTTTTGGAAATGTGATG 6060**

**33F_cps 6061 AGACAACATCCGACTCTAATGATAATAAATAGAATATTGTATTCTAAAGTTGATTTGATT 6120**

**||||||||||||||||||||||||||||||||||||||||||||||||||||||||||||**

**33F-1_cps 6061 AGACAACATCCGACTCTAATGATAATAAATAGAATATTGTATTCTAAAGTTGATTTGATT 6120**

**33F_cps 6121 ATATTATTGGGAGAGAAATCGAAAATAATGTTTCAACAACTAAGAATTTTAGACGAAAAA 6180**

**||||||||||||||||||||||||||||||||||||||||||||||||||||||||||||**

**33F-1_cps 6121 ATATTATTGGGAGAGAAATCGAAAATAATGTTTCAACAACTAAGAATTTTAGACGAAAAA 6180**

**33F_cps 6181 TTTAAAATAATTAGGAATGGTGTGGATGGATATTTATTTATTGAAAAAAATGAATTAAAT 6240**

**||||||||||||||||||||||||||||||||||||||||||||||||||||||||||||**

**33F-1_cps 6181 TTTAAAATAATTAGGAATGGTGTGGATGGATATTTATTTATTGAAAAAAATGAATTAAAT 6240**

**33F_cps 6241 AAAAAAATGAGCGATCTACCCATAAATATTATTTTTTTTAGTAATATGATTAGAGAAAAA 6300**

**||||||||||||||||||||||||||||||||||||||||||||||||||||||||||||**

**33F-1_cps 6241 AAAAAAATGAGCGATCTACCCATAAATATTATTTTTTTTAGTAATATGATTAGAGAAAAA 6300**

**33F_cps 6301 GGTTACGAAATTTTATTAGAAGTTGCTAAGAAGATGGTGGGGGATGAGAAATATCACTTT 6360**

**|||||:|||||||||||||||||||||||:||||||::||::||||||||||||||||||**

**33F-1_cps 6301 GGTTATGAAATTTTATTAGAAGTTGCTAAAAAGATGAGGGAAGATGAGAAATATCACTTT 6360**

**33F_cps 6361 TATTTTTCTGGAAAATTTCAGGATAATAATTTAAAGACTAGGTTTATTAATGAAATTTAT 6420**

**||||||||||||||||||||||||||||||||||||||||||||||||||||||||||||**

**33F-1_cps 6361 TATTTTTCTGGAAAATTTCAGGATAATAATTTAAAGACTAGGTTTATTAATGAAATTTAT 6420**

**33F_cps 6421 AGTATGAATAATGTAACATATTTAGATGGTGTATATGGTAGTGATAAGAAAAAGTTATTG 6480**

**|||||||||||||||||||||||||||||:||||||||||||||||||||||||||||||**

**33F-1_cps 6421 AGTATGAATAATGTAACATATTTAGATGGCGTATATGGTAGTGATAAGAAAAAGTTATTG 6480**

**33F_cps 6481 CAAAAAATGCATTACTTTGTTTTACCATCGTATTATAAAGATGAAACTTTACCTATCAGT 6540**

**|||||||||||||||||||||||||||||||||||||||||:||||||||||||||||||**

**33F-1_cps 6481 CAAAAAATGCATTACTTTGTTTTACCATCGTATTATAAAGACGAAACTTTACCTATCAGT 6540**

**33F_cps 6541 ATGTTAGAAGCAATGGCAAATGGTCTCTATATTATTGTAAGCGATGTGGGAGTTGTGTCA 6600**

**|||||||||||||||||||||||||||||||||||||||||||||||||||||||||||:**

**33F-1_cps 6541 ATGTTAGAAGCAATGGCAAATGGTCTCTATATTATTGTAAGCGATGTGGGAGTTGTGTCT 6600**

**33F_cps 6601 GAGGTGATTAATAAAGAAACTGCTAGTCTTATTGAAATGATAAACGAAGAAACAGCAGAC 6660**

**||||||||||||||||||||||||||||||||||||||||||||||||||||||||||||**

**33F-1_cps 6601 GAGGTGATTAATAAAGAAACTGCTAGTCTTATTGAAATGATAAACGAAGAAACAGCAGAC 6660**

**33F_cps 6661 AGTATCATAGAAATTATTAATCAGACTTCAAATAAACTTAATGAGTTAGATTTTAATGTA 6720**

**|||||:||||||||||||:|||||||||||||||||||||||||||||||||||||||||**

**33F-1_cps 6661 AGTATTATAGAAATTATTGATCAGACTTCAAATAAACTTAATGAGTTAGATTTTAATGTA 6720**

**33F_cps 6721 TCAAAATATAAGCAAGAGTTGTTAAATGAAAATATACAAGCATCAATTTATCAGCAATTA 6780**

**||||||||||||||||||||||||||||||||||||||||||||||||||||||||||||**

**33F-1_cps 6721 TCAAAATATAAGCAAGAGTTGTTAAATGAAAATATACAAGCATCAATTTATCAGCAATTA 6780**

**33F_cps 6781 GAGAGGATAGCAAATTGACGAAGAAAAAAAATACTGGAAAAATATTAACAGTAGTTGTAC 6840**

**||||||||||||||||||||:|||||||||||||||||||||||||||||||||||||||**

**33F-1_cps 6781 GAGAGGATAGCAAATTGACGGAGAAAAAAAATACTGGAAAAATATTAACAGTAGTTGTAC 6840**

**33F_cps 6841 CATCATATAATGCAGAAAATTATTTACAAGAGACGATGCCGACGATACTTTCTGCTAAGA 6900**

**||||||||||||||||||||||||||||||||||||||||||||||||||||||||||||**

**33F-1_cps 6841 CATCATATAATGCAGAAAATTATTTACAAGAGACGATGCCGACGATACTTTCTGCTAAGA 6900**

**33F_cps 6901 ATATAGAGAGGGTAGAATTACTGATTGTAAACGATGGTTCTACAGACAGAACCGAAGAAA 6960**

**||||||||||||||||||||||||||||||||||||||||||||||||:|||||||||||**

**33F-1_cps 6901 ATATAGAGAGGGTAGAATTACTGATTGTAAACGATGGTTCTACAGACAAAACCGAAGAAA 6960**

**33F_cps 6961 TAGCAAGACAATTCGAGAGAGAATATGAGGGAATTGTTAGAGTTATAAGTAAGGAAAACT 7020**

**|||||||||||||||||||||||||||||||||||||:|||||||||||||||||||||:**

**33F-1_cps 6961 TAGCAAGACAATTCGAGAGAGAATATGAGGGAATTGTCAGAGTTATAAGTAAGGAAAACG 7020**

**33F_cps 7021 GTGGTCACGGTTCGGCAGTAAATGCGGGGATTGAGAATGCTGTTGGTAATTACTTTAAAG 7080**

**||||||||||||||||||||||||||||:|||||:|||||||||||||||||||||||||**

**33F-1_cps 7021 GTGGTCACGGTTCGGCAGTAAATGCGGGAATTGAAAATGCTGTTGGTAATTACTTTAAAG 7080**

**33F_cps 7081 TGGTAGATGCAGATGATTGGGTGAACACCAATAATTTAGAAGATTTGATAGTGTTTCTAT 7140**

**||||||||||||||||||||||||||||||||||||||||||||||||||||||||||||**

**33F-1_cps 7081 TGGTAGATGCAGATGATTGGGTGAACACCAATAATTTAGAAGATTTGATAGTGTTTCTAT 7140**

**33F_cps 7141 CTGAGGTGGATGTGGATCAAGTTTTATCACCATATGATAAAATTTTTGTGAATTATCGAG 7200**

**|||||||||||||||||||||||||||||||:||||||||||||||||||||||||||||**

**33F-1_cps 7141 CTGAGGTGGATGTGGATCAAGTTTTATCACCGTATGATAAAATTTTTGTGAATTATCGAG 7200**

**33F_cps 7201 GTGATATAGAGCGTGAAGAAGAGTGCAACGAGTTTTCGCAAGTTGAGAACGAGGTGATCT 7260**

**||||||||||||||||||||||||||||||||||||||||||||||||||||||||||||**

**33F-1_cps 7201 GTGATATAGAGCGTGAAGAAGAGTGCAACGAGTTTTCGCAAGTTGAGAACGAGGTGATCT 7260**

**33F_cps 7261 ATTCTGCAGAAGAATTCTATACACGAATTAAACAAACGGTAGGAATGCATTCCATTACTG 7320**

**||||||||||||||||||||||||||||||||||||||||||||||||||||||||||||**

**33F-1_cps 7261 ATTCTGCAGAAGAATTCTATACACGAATTAAACAAACGGTAGGAATGCATTCCATTACTG 7320**

**33F_cps 7321 TGAAAACGAGTCTTTTGCAAGAGAACAATATTCGGCTGTCTGAAAAAATGTTCTATGTAG 7380**

**||||||||||||||||||||||||||||||||||||||||||||||||||||||||||||**

**33F-1_cps 7321 TGAAAACGAGTCTTTTGCAAGAGAACAATATTCGGCTGTCTGAAAAAATGTTCTATGTAG 7380**

**33F_cps 7381 ATATGGAATATATTGTTTATATGCTTCCATATGTAAAAAAGGTTGTTTTATTTGATAAAA 7440**

**||||||||||||||||||||||:||||||||||||||||:||||||||||||||||||||**

**33F-1_cps 7381 ATATGGAATATATTGTTTATATACTTCCATATGTAAAAAGGGTTGTTTTATTTGATAAAA 7440**

**33F_cps 7441 GTATCTATAGATATCGTCTAGGAACAGAGACACAAAGCATTAGTATGGCGAGTTATATTA 7500**

**||||:|||||||||||||||||||||||||||||||||:|||||||||||||||||||||**

**33F-1_cps 7441 GTATTTATAGATATCGTCTAGGAACAGAGACACAAAGCGTTAGTATGGCGAGTTATATTA 7500**

**33F_cps 7501 AAAATCGTGATATGCATAAACAAGTGATCTATCATTTGGTTGATTTTTATAATCAAATGA 7560**

**||||||||||||||||||||||||||||||||||||||||||||||||||||||||||||**

**33F-1_cps 7501 AAAATCGTGATATGCATAAACAAGTGATCTATCATTTGGTTGATTTTTATAATCAAATGA 7560**

**33F_cps 7561 GATCTAGCGCTGTTTTAAGGAGAATAACATGGAAATTGATTTTAAATTTAATAAGACAGC 7620**

**||||||||||||||||||:|||||||||||||||||||||||||||||||||||||||||**

**33F-1_cps 7561 GATCTAGCGCTGTTTTAAAGAGAATAACATGGAAATTGATTTTAAATTTAATAAGACAGC 7620**

**33F_cps 7621 AATGGATAATTTATTTTAATTTATCTAAAAAAGAGGGGAAAAACAGCGAGTGTTTTGAAT 7680**

**||||||||||||||||||||||||||||||||||||||||||||||||||||||||||||**

**33F-1_cps 7621 AATGGATAATTTATTTTAATTTATCTAAAAAAGAGGGGAAAAACAGCGAGTGTTTTGAAT 7680**

**33F_cps 7681 TTGATAATTGGCTAATAAAAGAAGGACGGATAAAAAAAATACCACTATACTTTTTTAAAG 7740**

**||||||||||||||||||||||||||||||||||||||||||||||||||||||||||||**

**33F-1_cps 7681 TTGATAATTGGCTAATAAAAGAAGGACGGATAAAAAAAATACCACTATACTTTTTTAAAG 7740**

**33F_cps 7741 CTGTGAAATATATCAGATTTAAGGTGAAATATTTTCTGGGGATAAGAAAATAATATGAGA 7800**

**||||||||||||||||||||||||||||||||||||||||||||||:||||||||||||:**

**33F-1_cps 7741 CTGTGAAATATATCAGATTTAAGGTGAAATATTTTCTGGGGATAAGGAAATAATATGAGG 7800**

**33F_cps 7801 AAAATAGGAAAAGTAATAAACGAGTATTTTGTATTAAGAAAATCATTTACTCCTGCTATT 7860**

**|||||||||||||||||||||||||||||||:||||||||||||||||||||||||||||**

**33F-1_cps 7801 AAAATAGGAAAAGTAATAAACGAGTATTTTGCATTAAGAAAATCATTTACTCCTGCTATT 7860**

**33F_cps 7861 GCTCGGAATAAATTATTTGAAAAGTTTTGGGGACGGATAGGGAATTATAAAATTTTCAAT 7920**

**||||||||||||||||||||||||||||||||||||||||||||||||||||||||||||**

**33F-1_cps 7861 GCTCGGAATAAATTATTTGAAAAGTTTTGGGGACGGATAGGGAATTATAAAATTTTCAAT 7920**

**33F_cps 7921 AATATAGCTAGTAATTTTTATCAATACAAACATGAAACAATCATAAATTTTTTGGAAAAA 7980**

**||||||||||||:|||||||||||||||||||||||||||||||||||||||||||||||**

**33F-1_cps 7921 AATATAGCTAGTGATTTTTATCAATACAAACATGAAACAATCATAAATTTTTTGGAAAAA 7980**

**33F_cps 7981 GATTTTAGCCAATTTTTAAAATCCTATAACTTTAAAGAGGTATCGCACAAGGAGATAGAA 8040**

**||||||||||||||||||||||||||||||||||||||||||||||||||||||||||||**

**33F-1_cps 7981 GATTTTAGCCAATTTTTAAAATCCTATAACTTTAAAGAGGTATCGCACAAGGAGATAGAA 8040**

**33F_cps 8041 CAAAGAAAAATCTTCTCTATGTGGATACAAGGATACGAAAGTGCTCCTAAGTTGGTTCAG 8100**

**||||||||||||||||||||||||||||||||||||||||||||||||||||||||||||**

**33F-1_cps 8041 CAAAGAAAAATCTTCTCTATGTGGATACAAGGATACGAAAGTGCTCCTAAGTTGGTTCAG 8100**

**33F_cps 8101 AAGACGATTGATAGCCAAAGGAAATATGCAGAAAAATACGGATATAAATTTGTTTTTTTG 8160**

**||||||||||||||||||||||||||||||||||||||||||||||||||||||||||||**

**33F-1_cps 8101 AAGACGATTGATAGCCAAAGGAAATATGCAGAAAAATACGGATATAAATTTGTTTTTTTG 8160**

**33F_cps 8161 GATGAAAACAATATCCGTGAATATGTGACACTTCCATCAGAGATAGTAGAAAAATATGAG 8220**

**|||:||||||||||||||||||||||||||||||||||||||||||||||||||||||||**

**33F-1_cps 8161 GATAAAAACAATATCCGTGAATATGTGACACTTCCATCAGAGATAGTAGAAAAATATGAG 8220**

**33F_cps 8221 AATGGAACTATAGATTTTATAAAATATTCGGATGTTGTAAGGGGAACATTGCTTTCAAAA 8280**

**||||||||||||||||||||||||||||||||||||||||||||||||||||||||||||**

**33F-1_cps 8221 AATGGAACTATAGATTTTATAAAATATTCGGATGTTGTAAGGGGAACATTGCTTTCAAAA 8280**

**33F_cps 8281 TATGGAGGAGTGTGGTTAGATTCGACTATTTATGTTGATTCTTCACGAGAACTGAATTAT 8340**

**||||||||||||||||||||||||||||||||||||||||||||||||||||||||||||**

**33F-1_cps 8281 TATGGAGGAGTGTGGTTAGATTCGACTATTTATGTTGATTCTTCACGAGAACTGAATTAT 8340**

**33F_cps 8341 TTAAAAAAAGATTTTTATACTATTCGAGCAAAGACACATGAGAGAGTGCCCAAATATATC 8400**

**||||||||||||||||||||||||||||||||||||||||||||||||||||||||||||**

**33F-1_cps 8341 TTAAAAAAAGATTTTTATACTATTCGAGCAAAGACACATGAGAGAGTGCCCAAATATATC 8400**

**33F_cps 8401 GCAAATGGTAGGTGGTCTGCATTTTGTTTGTCAGGGGAAAAGCAAAACATAGTATTTGAT 8460**

**||||||||||||||||||||||||||||||||||||||||||||||||||||||||||||**

**33F-1_cps 8401 GCAAATGGTAGGTGGTCTGCATTTTGTTTGTCAGGGGAAAAGCAAAACATAGTATTTGAT 8460**

**33F_cps 8461 TTTTTAGAGAAATTTCATGTAGCATATTTTATGAAATACGATATAGTGCTAGATTATTTT 8520**

**||||||||||||||||||||||||||||||||||||||||||||||||||||||||||||**

**33F-1_cps 8461 TTTTTAGAGAAATTTCATGTAGCATATTTTATGAAATACGATATAGTGCTAGATTATTTT 8520**

**33F_cps 8521 TTAATTGACTATATTATAGAATTAGGTTATAGGACAAATGACTTGATTAGAAACTATATT 8580**

**||:|||||||||||||||||||||||||||||||||||||||||||||||||||||||||**

**33F-1_cps 8521 TTGATTGACTATATTATAGAATTAGGTTATAGGACAAATGACTTGATTAGAAACTATATT 8580**

**33F_cps 8581 GATAAAGTCGAAGAAAATAATCAGGAGTTGTTCTTTTTGGCAGACAATTTTTCTAACCAG 8640**

**||||||||||||||||||||||||||||||||||||||||||||||||||||||||||||**

**33F-1_cps 8581 GATAAAGTCGAAGAAAATAATCAGGAGTTGTTCTTTTTGGCAGACAATTTTTCTAACCAG 8640**

**33F_cps 8641 TATGATGAAAAAGAGTGGGCAGGAGTTTTATCAACAACTGCATTGTTTAAGTGTAGTTAT 8700**

**||||||||||||||||||||||||||||||||||||||||||||||||||||||||||||**

**33F-1_cps 8641 TATGATGAAAAAGAGTGGGCAGGAGTTTTATCAACAACTGCATTGTTTAAGTGTAGTTAT 8700**

**33F_cps 8701 AAGTGTCCGATAAATGAAGCGACAGGAACCTATTTTGATAGATTGATGAAGGGTGAACTG 8760**

**||||||||||||||||||||||||||||||||||||||||||||||||||||||||||||**

**33F-1_cps 8701 AAGTGTCCGATAAATGAAGCGACAGGAACCTATTTTGATAGATTGATGAAGGGTGAACTG 8760**

**33F_cps 8761 TAGCATAAAGAGAGGGAATGGTATGATTTCAGTTATTGTTCCAGTATACAATGTCGCGGA 8820**

**||||||||||||||||||||||||||||||||||||||||||||||||||||||||||||**

**33F-1_cps 8761 TAGCATAAAGAGAGGGAATGGTATGATTTCAGTTATTGTTCCAGTATACAATGTCGCGGA 8820**

**33F_cps 8821 TTATCTGCGCTTTGCGTTGGATAGTCTTTTGGAACAGACGTATAAAGATTTTGAGGTTAT 8880**

**|||||||||||||||||||||||||||||||||||||||||||||||||||||||:||||**

**33F-1_cps 8821 TTATCTGCGCTTTGCGTTGGATAGTCTTTTGGAACAGACGTATAAAGATTTTGAGATTAT 8880**

**33F_cps 8881 TCTAGTAAATGATGGATCCACTGATAATTCTGGGGAAATATGTGATGAATATGGGAAACT 8940**

**|||||||||||||||||||||||||||||||||:||||||||||||||||||||||||||**

**33F-1_cps 8881 TCTAGTAAATGATGGATCCACTGATAATTCTGGAGAAATATGTGATGAATATGGGAAACT 8940**

**33F_cps 8941 GTATGATAATATTCATGTTTTCCATAAGAAGAATGGCGGACTTTCGGATGCTAGAAATTT 9000**

**:||||||||||||:||||||||||||||||||||||||||||||||||||||||||||||**

**33F-1_cps 8941 ATATGATAATATTTATGTTTTCCATAAGAAGAATGGCGGACTTTCGGATGCTAGAAATTT 9000**

**33F_cps 9001 TGGTTTGGAGAAGAGTCGAGGAGAGTTTATAACTTTTCTTGATTCAGATGATTATTTTGA 9060**

**|||||||||||||||||||||||||||||||||:||||||||||||||||||||||||||**

**33F-1_cps 9001 TGGTTTGGAGAAGAGTCGAGGAGAGTTTATAACCTTTCTTGATTCAGATGATTATTTTGA 9060**

**33F_cps 9061 ACCGTATGCATTAGAATTGTTAATTACTATTCAAAAAAAATATGATGTAGATATCGTATC 9120**

**||||||||||||||||||||||||||||||||||||||||||||||||||||||||||||**

**33F-1_cps 9061 ACCGTATGCATTAGAATTGTTAATTACTATTCAAAAAAAATATGATGTAGATATCGTATC 9120**

**33F_cps 9121 AACTAAAGGGGGAATAACCTATTCTCATGATATTTATAGCAAAAAACTGATGGCTGAGGA 9180**

**|||||||||||:|||||||||||||||||||||||||||||||||||||||||:||||||**

**33F-1_cps 9121 AACTAAAGGGGTAATAACCTATTCTCATGATATTTATAGCAAAAAACTGATGGATGAGGA 9180**

**33F_cps 9181 CTACCTAACTGTAAAAATATTGACAAATAAAGAATTTTTGGCAGCTGTATATTATAACGA 9240**

**|||||||||||||||||||||||||||||||||||||||||||||||:||||||||||||**

**33F-1_cps 9181 CTACCTAACTGTAAAAATATTGACAAATAAAGAATTTTTGGCAGCTGCATATTATAACGA 9240**

**33F_cps 9241 TGAAATGACGGTATCTGCTTGGGGAAAATTGTATAAAAGAGATTTATTTAAAACGATTTT 9300**

**||||||||||||||||||||||||||||||||||||||||||||||||||||||||||||**

**33F-1_cps 9241 TGAAATGACGGTATCTGCTTGGGGAAAATTGTATAAAAGAGATTTATTTAAAACGATTTT 9300**

**33F_cps 9301 TCCAAAAGGAAAGATCTACGAGGATTTGTATGTTGTTGCAGAGCGTTTGTTGAATATTAA 9360**

**||||||||||||||||||||||||||||||||||||||||||||||||||||||||||||**

**33F-1_cps 9301 TCCAAAAGGAAAGATCTACGAGGATTTGTATGTTGTTGCAGAGCGTTTGTTGAATATTAA 9360**

**33F_cps 9361 AACAGTTGCTCATACCGATTTGCCTATATATCATTATTATCAGAGACAGGGAAGTATTGT 9420**

**||||||||||||||||||||||||||||||||||||||||||||||||||||||||||||**

**33F-1_cps 9361 AACAGTTGCTCATACCGATTTGCCTATATATCATTATTATCAGAGACAGGGAAGTATTGT 9420**

**33F_cps 9421 TAATTCAACATTTTCGGATAGACAGTATGATTTTTTTGATGCGATTGATCATAATGAAGC 9480**

**||||||||||||||||||||||||||||||||||||||||||||||||||||||||||||**

**33F-1_cps 9421 TAATTCAACATTTTCGGATAGACAGTATGATTTTTTTGATGCGATTGATCATAATGAAGC 9480**

**33F_cps 9481 CATAATAAAAAAATTCTATTGTGGAGATAAGGAATTGTTAGCAGCCTTAAATGCTAAAAG 9540**

**||||||||||||:||||||||||||||||||||||||:||||||||||||||||||||||**

**33F-1_cps 9481 CATAATAAAAAAGTTCTATTGTGGAGATAAGGAATTGCTAGCAGCCTTAAATGCTAAAAG 9540**

**33F_cps 9541 AGTAATTGGCTCCTTTATTTTGAGTAATAGCGCATTTTATAATAGTAAAAACGATATTAC 9600**

**||||||||||||||||||||||||||||||||||||||||||||||||||||||||||||**

**33F-1_cps 9541 AGTAATTGGCTCCTTTATTTTGAGTAATAGCGCATTTTATAATAGTAAAAACGATATTAC 9600**

**33F_cps 9601 TAAAATAATAAGAATAATTAAACCGTATTATTGGGAAGTAATAAAAAATAAAAAAATTCC 9660**

**||||||||||||||||||||||||||||||||||||||||||||||||||||||||||||**

**33F-1_cps 9601 TAAAATAATAAGAATAATTAAACCGTATTATTGGGAAGTAATAAAAAATAAAAAAATTCC 9660**

**33F_cps 9661 AATGAAAAGGAAAGTTCAATGTGTTTTGTTTCTGCTTTCCCCAAATTACTACTATAAAAT 9720**

**||||||||||||||||||||||||||||||||||||||||||||||||||||||||||||**

**33F-1_cps 9661 AATGAAAAGGAAAGTTCAATGTGTTTTGTTTCTGCTTTCCCCAAATTACTACTATAAAAT 9720**

**33F_cps 9721 AAAAGATAAGATGTTACAGAGAGGCAGGATATAGATGCATGTTAGATTAGATGGTTTGCT 9780**

**||||||||||||||||||||||||||||||||||||||||||||||||||||||||||||**

**33F-1_cps 9721 AAAAGATAAGATGTTACAGAGAGGCAGGATATAGATGCATGTTAGATTAGATGGTTTGCT 9780**

**33F_cps 9781 GGACTATATATTTCTATTTAGTGTGATTATTACTTGTAATACTATGTATTCAACTAGTCA 9840**

**||||||||||||||||||||||||||||||||||||||||||||||||||||||||||||**

**33F-1_cps 9781 GGACTATATATTTCTATTTAGTGTGATTATTACTTGTAATACTATGTATTCAACTAGTCA 9840**

**33F_cps 9841 AGGATTTGATGGACTAGGGAAATGGGCGACTCTGTTACTTGTGGTATCAGTTTTTCTGAA 9900**

**||||||||||||||||||||||||||||||||||||||||||||||||||||:|||||||**

**33F-1_cps 9841 AGGATTTGATGGACTAGGGAAATGGGCGACTCTGTTACTTGTGGTATCAGTTATTCTGAA 9900**

**33F_cps 9901 ATTGCTTATCTCTAGAATATCTATGAAGGCAATCAATGTGATTGTGTCGCGTTCTTTAAT 9960**

**||||||||||||||||||||||||||||||||||||||||||||||||||||||||||||**

**33F-1_cps 9901 ATTGCTTATCTCTAGAATATCTATGAAGGCAATCAATGTGATTGTGTCGCGTTCTTTAAT 9960**

**33F_cps 9961 ATTTATATTAATTATTCTACTCATAGTAATATTAAATGGTTTTAAGATTTCTGAGACAAG 10020**

**||||||||||||||||||||||||||||||||||||||||||||||||||||||||||||**

**33F-1_cps 9961 ATTTATATTAATTATTCTACTCATAGTAATATTAAATGGTTTTAAGATTTCTGAGACAAG 10020**

**33F_cps 10021 TTTCGTCTATTATTTTGTATTATTTCCGATTTTTATGATGATTTTGCAGATGTACTATGA 10080**

**||||||||||||||||||||||||||||||||||||||||||||||||||||||||||||**

**33F-1_cps 10021 TTTCGTCTATTATTTTGTATTATTTCCGATTTTTATGATGATTTTGCAGATGTACTATGA 10080**

**33F_cps 10081 TGTTAATGAAATCGCAAATCTGATACGGAAATTTGTTCGTATAATATTTCTTTTAGCAAT 10140**

**||||||||||||||||||||||||||||||||||||||||||||||||||||||||||||**

**33F-1_cps 10081 TGTTAATGAAATCGCAAATCTGATACGGAAATTTGTTCGTATAATATTTCTTTTAGCAAT 10140**

**33F_cps 10141 TGGCTCTCTCCTATTTTGGCTTATTGGTAGTGTATTTCATATTATATCCCCAACGGTTTA 10200**

**||||||||||||||||||||||||||||||||||||||||||||||||||||||||||||**

**33F-1_cps 10141 TGGCTCTCTCCTATTTTGGCTTATTGGTAGTGTATTTCATATTATATCCCCAACGGTTTA 10200**

**33F_cps 10201 TGTGTTGAATTATTGGAATGGTGGGGGAATAGTAGAAGGGTACTATAATCTTCATTTTGA 10260**

**||||||||||||||||||||||||||||||||||||||||||||||||||||||||||||**

**33F-1_cps 10201 TGTGTTGAATTATTGGAATGGTGGGGGAATAGTAGAAGGGTACTATAATCTTCATTTTGA 10260**

**33F_cps 10261 AGCACAAAAAATAGAGATTTTGGGGGCGATACTGATAAGAAATACGGGGATTTTTGCTGA 10320**

**||||||||||||||||||||||||||||||||||||||||||||||||||||||||||||**

**33F-1_cps 10261 AGCACAAAAAATAGAGATTTTGGGGGCGATACTGATAAGAAATACGGGGATTTTTGCTGA 10320**

**33F_cps 10321 AGCACCTATGTGGAGTTTGGTATTGAGCCTTGCATTGATATTTCAAACGCTTCATATAAA 10380**

**||||||||||||||||||||||||||||||||||||||||||||||||||||||||||||**

**33F-1_cps 10321 AGCACCTATGTGGAGTTTGGTATTGAGCCTTGCATTGATATTTCAAACGCTTCATATAAA 10380**

**33F_cps 10381 AAAATGGAATTTCACAACTTGGACTCTTATTATTACAATTATGACAACTACGTCAACAAC 10440**

**||||||||||||||||||||||||||||||||||||||||||||||||||||||||||||**

**33F-1_cps 10381 AAAATGGAATTTCACAACTTGGACTCTTATTATTACAATTATGACAACTACGTCAACAAC 10440**

**33F_cps 10441 AGGGGTTTATATAATAGGTTTGATTTTCCTATATGTTTTATTTTCAAAAACAAGTGGTGT 10500**

**||||||||||||||||||||||||||||||||||||||||||||||||||||||||||||**

**33F-1_cps 10441 AGGGGTTTATATAATAGGTTTGATTTTCCTATATGTTTTATTTTCAAAAACAAGTGGTGT 10500**

**33F_cps 10501 GAAGAGATATGTTTCTAGTTTATTTATTTTAGCGATTATATGTTGTTTTTCAATATTATG 10560**

**||||||||||||||||||||||||||||||||||||||||||||||||||||||||||||**

**33F-1_cps 10501 GAAGAGATATGTTTCTAGTTTATTTATTTTAGCGATTATATGTTGTTTTTCAATATTATG 10560**

**33F_cps 10561 GGATAATAAATCTGGAACTGGTTCAGCAACTATACGCTTTGATGATTATAAGGCTGGTTT 10620**

**||||||||||||||||||||||||||||||||||||||||||||||||||||||||||||**

**33F-1_cps 10561 GGATAATAAATCTGGAACTGGTTCAGCAACTATACGCTTTGATGATTATAAGGCTGGTTT 10620**

**33F_cps 10621 TTTGGCATGGCAGAAAAGTCCTATTTGGGGATTAGGTATTTCAGACGGTCTTAGAACCAT 10680**

**||||||||||||||||||||||||||||||||||||||||||||||||||||||||||||**

**33F-1_cps 10621 TTTGGCATGGCAGAAAAGTCCTATTTGGGGATTAGGTATTTCAGACGGTCTTAGAACCAT 10680**

**33F_cps 10681 TGAACAACATATGGATAGAACTGTTAGATATAATCTTGGGTATAGCAATAGCTTTTTTGT 10740**

**||||||||||||||||||||||||||||||||||||||||||||||||||||||||||||**

**33F-1_cps 10681 TGAACAACATATGGATAGAACTGTTAGATATAATCTTGGGTATAGCAATAGCTTTTTTGT 10740**

**33F_cps 10741 TGTTTTAGCTCAAGGAGGGATAATGTTGGCTTCGTATTACTTTTATCCAGTAATTAAAAT 10800**

**||||||||||||||||||||||||||||||||||||||||||||||||||||||||||||**

**33F-1_cps 10741 TGTTTTAGCTCAAGGAGGGATAATGTTGGCTTCGTATTACTTTTATCCAGTAATTAAAAT 10800**

**33F_cps 10801 TATTTTGAATAAATTTAGTTCGAATGATTTGAAGTTTTCTGCGCTATTAATAATTTTCCT 10860**

**||||||||||||||||||||||||||||||||||||||||||||||||||||||||||||**

**33F-1_cps 10801 TATTTTGAATAAATTTAGTTCGAATGATTTGAAGTTTTCTGCGCTATTAATAATTTTCCT 10860**

**33F_cps 10861 TATGATAACTACTATATTTATAGAGACCTATATGTTTTTATTTGTCATTTCTTTATACTA 10920**

**||||||||||||||||||||||||||||||||||||||||||||||||||||||||||||**

**33F-1_cps 10861 TATGATAACTACTATATTTATAGAGACCTATATGTTTTTATTTGTCATTTCTTTATACTA 10920**

**33F_cps 10921 TTCTCTTGATTTTGGGGACGATAGAGATTGTCATGAGAAACAGTACATTACTAATTAATA 10980**

**||||||||||||||||||||||||||||||||||||||||||||||||||||||||||||**

**33F-1_cps 10921 TTCTCTTGATTTTGGGGACGATAGAGATTGTCATGAGAAACAGTACATTACTAATTAATA 10980**

**33F_cps 10981 ATAAAGGTGTGAACAGAAATAAGAAGAAATGAAAATACTAAAAAACTATGCCTACAATCT 11040**

**|||||||||||||||||||||||||||||||||||||||||||||:||||||||||||||**

**33F-1_cps 10981 ATAAAGGTGTGAACAGAAATAAGAAGAAATGAAAATACTAAAAAATTATGCCTACAATCT 11040**

**33F_cps 11041 TTCTTATCAATTGTTGGTGATCATACTCCCTATCATTACGACTCCCTATGTAACGAGGGT 11100**

**||||||||||||||||||||||||||||||||||||||||||||||||||||||||||||**

**33F-1_cps 11041 TTCTTATCAATTGTTGGTGATCATACTCCCTATCATTACGACTCCCTATGTAACGAGGGT 11100**

**33F_cps 11101 TTTTAGTTCTGACGATTTAGGAACGTATGGCTACTTTAGCTCCATTGTTACCTATTTTAC 11160**

**||||||||||||||||||||||||||||||||||||||||||||||||||||||||||||**

**33F-1_cps 11101 TTTTAGTTCTGACGATTTAGGAACGTATGGCTACTTTAGCTCCATTGTTACCTATTTTAC 11160**

**33F_cps 11161 CTTGCTTGCAACTCTTGGTGTTGCCAACTACGGTACCAAAGAGATTTCAGCACATCGTAA 11220**

**||||||||||||||||||||||||||||||||||||||||||||||||||||||||||||**

**33F-1_cps 11161 CTTGCTTGCAACTCTTGGTGTTGCCAACTACGGTACCAAAGAGATTTCAGCACATCGTAA 11220**

**33F_cps 11221 GGAAATTGGGAAGAATTTCTGGGGAATTTATTCTCTCCAGTTTGGTGCAACTTGGCTATC 11280**

**||||||||||||||||||||||||||||||||||||||||||||||||||||||||||||**

**33F-1_cps 11221 GGAAATTGGGAAGAATTTCTGGGGAATTTATTCTCTCCAGTTTGGTGCAACTTGGCTATC 11280**

**33F_cps 11281 CATTTTGCTTTATCTTGCCCTTTGTTTCTTATTTACTTCAATGCAAAATCCGGTAGCTTA 11340**

**||||||||||||||||||||||||||||||||||||||||||||||||||||||||||||**

**33F-1_cps 11281 CATTTTGCTTTATCTTGCCCTTTGTTTCTTATTTACTTCAATGCAAAATCCGGTAGCTTA 11340**

**33F_cps 11341 TATATTGGGATTAAGTTTAGTGTCAAAAGGTTTGGATATTTCTTGGTTATTTCAAGGTTT 11400**

**||||||||||||||||||||||||||||||||||||||||||||||||||||||||||||**

**33F-1_cps 11341 TATATTGGGATTAAGTTTAGTGTCAAAAGGTTTGGATATTTCTTGGTTATTTCAAGGTTT 11400**

**33F_cps 11401 GGAGGATTTTAGAAAGATTACAGTTCGGAACATCACTGTTAAGTTAGTTGGTGCAATCTC 11460**

**||||||||||||||||||||||||||||||||||||||||||||||||||||||||||||**

**33F-1_cps 11401 GGAGGATTTTAGAAAGATTACAGTTCGGAACATCACTGTTAAGTTAGTTGGTGCAATCTC 11460**

**33F_cps 11461 TATTTTCCTATTTGTAAAATCAGCTAATGACTTATACTTATATGTATTTCTTCTTACCAT 11520**

**||||||||||||||||||||||||||||||||||||||||||||||||||||||||||||**

**33F-1_cps 11461 TATTTTCCTATTTGTAAAATCAGCTAATGACTTATACTTATATGTATTTCTTCTTACCAT 11520**

**33F_cps 11521 TTTCGAATTGTTAGGGCAATTAAGCATGTGGTTACCCGCAAGACAATTTATTGGTAAATC 11580**

**||||||||||||||||||||||||||||||||||||||||||||||||||||||||||||**

**33F-1_cps 11521 TTTCGAATTGTTAGGGCAATTAAGCATGTGGTTACCCGCAAGACAATTTATTGGTAAATC 11580**

**33F_cps 11581 ATATTTTGATTGGAAATACGCTAAGAAGCATTTGAAACCAGTTATCTTGTTATTCTTGCC 11640**

**||||||||||||||||||||||||||||||||||||||||||||||||||||||||||||**

**33F-1_cps 11581 ATATTTTGATTGGAAATACGCTAAGAAGCATTTGAAACCAGTTATCTTGTTATTCTTGCC 11640**

**33F_cps 11641 ACAGATTGCCATCTCGCTTTATGTGACTTTGGACCGTACGATGCTGGGGGTGTTGGCTTC 11700**

**||||||||||||||||||||||||||||||||||||||||||||||||||||||||||||**

**33F-1_cps 11641 ACAGATTGCCATCTCGCTTTATGTGACTTTGGACCGTACGATGCTGGGGGTGTTGGCTTC 11700**

**33F_cps 11701 GACAAAGGATGTTGGAATTTATGATCAAGCTTTGAAGCTTGTTAATATTTTACTGACTTT 11760**

**||||||||||||||||||||||||||||||||||||||||||||||||||||||||||||**

**33F-1_cps 11701 GACAAAGGATGTTGGAATTTATGATCAAGCTTTGAAGCTTGTTAATATTTTACTGACTTT 11760**

**33F_cps 11761 GGTTACCTCACTGGGGAGTGTTATGTTGCCACGAGTATCTAGCCTTTTATCGTCTGGGGA 11820**

**||||||||||||||||||||||||||||||||||||||||||||||||||||||||||||**

**33F-1_cps 11761 GGTTACCTCACTGGGGAGTGTTATGTTGCCACGAGTATCTAGCCTTTTATCGTCTGGGGA 11820**

**33F_cps 11821 TTACAAAGCCGTTAATAAGATGCACGAGATGTCTTTTTTAATCTATAACTTGGTGATTTT 11880**

**|||:||||||||||||||||||||||||||||||||||||||||||||||||||||||||**

**33F-1_cps 11821 TTATAAAGCCGTTAATAAGATGCACGAGATGTCTTTTTTAATCTATAACTTGGTGATTTT 11880**

**33F_cps 11881 CCCAATCATAGCAGGAATGTTGATTGTCAACGATGATTTTGTTCGTTTCTTCCTTGGGCA 11940**

**||||||||||||||||||||||||||||||||||||||||||||||||||||||||||||**

**33F-1_cps 11881 CCCAATCATAGCAGGAATGTTGATTGTCAACGATGATTTTGTTCGTTTCTTCCTTGGGCA 11940**

**33F_cps 11941 AAATTTTCAAGATGCTCGCTATGCGATTGCTATTATGACCTTCCGTATGTTCTTTATTGG 12000**

**||||||||||||||||||||||||||||||||||||||||||||||||||||||||||||**

**33F-1_cps 11941 AAATTTTCAAGATGCTCGCTATGCGATTGCTATTATGACCTTCCGTATGTTCTTTATTGG 12000**

**33F_cps 12001 TTGGACCAATATAATGGGAATTCAAATCTTGATTCCGCACAATAAAAATAAGGAATTTAT 12060**

**||||||||||||||||||||||||||||||||||||||||||||||||||||||||||||**

**33F-1_cps 12001 TTGGACCAATATAATGGGAATTCAAATCTTGATTCCGCACAATAAAAATAAGGAATTTAT 12060**

**33F_cps 12061 GGTTTCAACGACAGTTCCCGCTATTCTGAGTGTGGGATTGAATTTGTTGCTTCTTCCCCA 12120**

**||||||||||||||||||||||||||||||||||||||||||||||||||||||||||||**

**33F-1_cps 12061 GGTTTCAACGACAGTTCCCGCTATTCTGAGTGTGGGATTGAATTTGTTGCTTCTTCCCCA 12120**

**33F_cps 12121 ATTTGGTTATATAGGTGCTGCGATTGTTTCAGTTTTGACTGAGGCGTTTGTATGGGGGAT 12180**

**||||||||||||||||||||||||||||||||||||||||||||||||||||||||||||**

**33F-1_cps 12121 ATTTGGTTATATAGGTGCTGCGATTGTTTCAGTTTTGACTGAGGCGTTTGTATGGGGGAT 12180**

**33F_cps 12181 TCAGTTATTCTATACACGTTTTTATTTAAAAGAAGTTCCTATTATCGGTTCAATGACAAA 12240**

**||||||||||||||||||||||||||||||||||||||||||||||||||||||||||||**

**33F-1_cps 12181 TCAGTTATTCTATACACGTTTTTATTTAAAAGAAGTTCCTATTATCGGTTCAATGACAAA 12240**

**33F_cps 12241 AACTGCACTTGCATCTGCTGTTATGTATGGTCTCTTGCTAGGTTCAAAAACATCTATACA 12300**

**||||||||||||||||||||||||||||||||||||||||||||||||||||||||||||**

**33F-1_cps 12241 AACTGCACTTGCATCTGCTGTTATGTATGGTCTCTTGCTAGGTTCAAAAACATCTATACA 12300**

**33F_cps 12301 TTTTTCGCCAACCATAAATGTTTTAATATTTTCGGTGCTAGGCGGAATTATCTATCTTTT 12360**

**||||||||||||||||||||||||||||||||||||||||||||||||||||||||||||**

**33F-1_cps 12301 TTTTTCGCCAACCATAAATGTTTTAATATTTTCGGTGCTAGGCGGAATTATCTATCTTTT 12360**

**33F_cps 12361 TGCAATTCTATCTCTGAAAGTGGTAGATGTGAAAGAATTAAAACAAATAATCAGGAAAAA 12420**

**||||||||||||||||||||||||||||||||||||||||||||||||||||||||||||**

**33F-1_cps 12361 TGCAATTCTATCTCTGAAAGTGGTAGATGTGAAAGAATTAAAACAAATAATCAGGAAAAA 12420**

**33F_cps 12421 TTAGAATGAGCAAATTTCGAAATATTAACTTAGATTTACTAAAAGTGCTTGCATGTGTTG 12480**

**||||||||||||||||||||||||||||||||||||||||||||||||||||||||||||**

**33F-1_cps 12421 TTAGAATGAGCAAATTTCGAAATATTAACTTAGATTTACTAAAAGTGCTTGCATGTGTTG 12480**

**33F_cps 12481 GAGTCGTTCTACTTCATACAACTATGGGTGGGTTTAAAGAGACAGGTGCATGGAATTTTT 12540**

**||||||||||||||||||||||||||||||||||||||||||||||||||||||||||||**

**33F-1_cps 12481 GAGTCGTTCTACTTCATACAACTATGGGTGGGTTTAAAGAGACAGGTGCATGGAATTTTT 12540**

**33F_cps 12541 TGACATATTTATATTACTTAGGAACCTATTCTATCCCTCTATTTTTTATGGTCAATGGTT 12600**

**||||||||||||||||||||||||||||||||||||||||||||||||||||||||||||**

**33F-1_cps 12541 TGACATATTTATATTACTTAGGAACCTATTCTATCCCTCTATTTTTTATGGTCAATGGTT 12600**

**33F_cps 12601 ATTTATTGTTGGGGAAGAGAGAAATTACCTATTCCTACATATTGCAAAAAATAAAATGGC 12660**

**||||||||||||||||||||||||||||||||||||||||||||||||||||||||||||**

**33F-1_cps 12601 ATTTATTGTTGGGGAAGAGAGAAATTACCTATTCCTACATATTGCAAAAAATAAAATGGC 12660**

**33F_cps 12661 TCCTAATAACAGTGTCGTCATGGACTTTTATCGTTTGGCTGTTTAAAAGAGACTTTACAG 12720**

**||||||||||||||||||||||||||||||||||||||||||||||||||||||||||||**

**33F-1_cps 12661 TCCTAATAACAGTGTCGTCATGGACTTTTATCGTTTGGCTGTTTAAAAGAGACTTTACAG 12720**

**33F_cps 12721 AGAACTTAATTAAAAAAATTATAGGTTCTTTGATACAAAAGGGGTATTTCTTCCAGTTTT 12780**

**||||||||||||||||||||||||||||||||||||||||||||||||||||||||||||**

**33F-1_cps 12721 AGAACTTAATTAAAAAAATTATAGGTTCTTTGATACAAAAGGGGTATTTCTTCCAGTTTT 12780**

**33F_cps 12781 GGTTTTTCGGTGCACTCATACTTATCTATTTATGTTTGCCAATTTTGAGACAATTTCTAA 12840**

**|||||||:|||||||||||||||||:|||:||||||||||::||||||:|:|:||:||:|**

**33F-1_cps 12781 GGTTTTTTGGTGCACTCATACTTATTTATATATGTTTGCCTGTTTTGAAAAATTTCCTTA 12840**

**33F_cps 12841 ATTCAAAAAGAAGCTATTTATACAGTTTATCTTTATTGATGACTATTGGTTTGATTTTTG 12900**

**|||:||||||||::||||||||||:|:|||||:|||||:||::|||||||||||||||||**

**33F-1_cps 12841 ATTTAAAAAGAAATTATTTATACATTCTATCTGTATTGCTGGTTATTGGTTTGATTTTTG 12900**

**33F_cps 12901 AGTTATCAAATATCCTACTTCAAATGCCAATACAAACATATGTAATACAAACTTTTAGAT 12960**

**|||||:|:|||||::||:|||||||||||::||||||||||||:||||||||:|||||||**

**33F-1_cps 12901 AGTTAGCGAATATTGTATTTCAAATGCCAGCACAAACATATGTTATACAAACCTTTAGAT 12960**

**33F_cps 12961 TATGGACGTGGTTTTTTTACTATCTTTTAGGTGGTTATATAGCGCAATTCACTATAGAAG 13020**

**|||||||:||||||||||||||:|||||||||||||:||||||||||||:|::|::||::**

**33F-1_cps 12961 TATGGACTTGGTTTTTTTACTACCTTTTAGGTGGTTTTATAGCGCAATTTAACAAGGATA 13020**

**33F_cps 13021 AAATCGAATCAAGGTTTAAGAATTGGATGAAAATAGTTAGCATACTTTTGTTATTGATTT 13080**

**::||:::|:::||||||||||::|||||||||:|||:||:::||||:||:|||||:||||**

**33F-1_cps 13021 TTATTAGAAATAGGTTTAAGATATGGATGAAAGTAGCTACTGTACTCTTATTATTAATTT 13080**

**33F_cps 13081 CACCAATAATATTATTTTTCATAGCGAAGACTATATACCATAATCTATTTGCTGAATACT 13140**

**|||||:||||||||||||||:||||||||||:|::|||||||||||:|||||||||||:|**

**33F-1_cps 13081 CACCATTAATATTATTTTTCTTAGCGAAGACCACTTACCATAATCTTTTTGCTGAATATT 13140**

**33F_cps 13141 TTTATGATACTTTATTTGTAAAAGTCAGTAC-TTTAGGAATTTTTCTAACTATCC-TCAT 13198**

**|||||||||:|||||||||||||||::|||::|||||||||||||||||||||:::||::**

**33F-1_cps 13141 TTTATGATATTTTATTTGTAAAAGT-TGTAAGTTTAGGAATTTTTCTAACTAT-ATTCTC 13198**

**33F_cps 13199 GCTTACTTTGAATGAAAACCGAAGAGAATCGATTGTTTCCCTTTCTAATCAAACAATGGG 13258**

**:|||:|:||||||:||:|::::|:::|||:||||:|||::||||||||:|||||:|||||**

**33F-1_cps 13199 ACTTGCATTGAATCAAGATATCAACAAATGGATTATTTTTCTTTCTAACCAAACTATGGG 13258**

**33F_cps 13259 GGTTTTCATAATACATACTTATATTATGAAAGTGTGGGAAAAAGTGC-TTGGTTTTAATT 13317**

**:||:||:||||||||:|||||||||||||||||||||||||||:|:::|||||||||:||**

**33F-1_cps 13259 TGTCTTTATAATACACACTTATATTATGAAAGTGTGGGAAAAACT-ATTTGGTTTTAGTT 13317**

**33F_cps 13318 TTGTAGGAGCATATTTACTTTTTGCTCTATTTACTTTAAGTGTTAGTTTTATTATTGTTG 13377**

**||||||||||||||||||||||||||:||||||||||||||:|||||||||||||||:||**

**33F-1_cps 13318 TTGTAGGAGCATATTTACTTTTTGCTATATTTACTTTAAGTATTAGTTTTATTATTGCTG 13377**

**33F_cps 13378 GGATGTTAATGAAGATTCCTTACTTCAATCGAATCGTCAAATTATAAAAAGGAGAACAAG 13437**

**|:|||||||||||:||||||||:|||||||||||:||||||||||||||||||||||||:**

**33F-1_cps 13378 GAATGTTAATGAAAATTCCTTATTTCAATCGAATTGTCAAATTATAAAAAGGAGAACAAA 13437**

**33F_cps 13438 ATGTACGATTATCTTATTGTCGGTGCTGGTTTGTCAGGAGCAATTTTTGCTTATGAGGCG 13497**

**||||||||:|||||:||:||||||||:||||||||:|||||:|||||||||:|:||:||:**

**33F-1_cps 13438 ATGTACGACTATCTAATCGTCGGTGCGGGTTTGTCTGGAGCTATTTTTGCTCACGAAGCT 13497**

**33F_cps 13498 ACCAAGCGTGGAAAAAAAGTAAAAGTGATTGATAAACG-TAACCACATTGGTGGGAATAT 13556**

**||:||:||||||||||||||||||||||||||||||||:::|:||||||||:|||||:||**

**33F-1_cps 13498 ACAAAACGTGGAAAAAAAGTAAAAGTGATTGATAAACGCG-ATCACATTGGAGGGAACAT 13556**

**33F_cps 13557 CTACTGTGAGAATGTAGAAGGAGTTAATGTTCATAAATATGGTGCCCATATCTTTCATAC 13616**

**|||||||||:|||||||||||::|:|||||:|||||||||||||||||||||||:|||||**

**33F-1_cps 13557 CTACTGTGAAAATGTAGAAGGTATCAATGTCCATAAATATGGTGCCCATATCTTCCATAC 13616**

**33F_cps 13617 TTCTAATAAGAAAGTTTGGGATTATGTTAATCAATTTGCTGAATTTAACAACTACGTCAA 13676**

**:||:||:||:|||||||||||:|||||:||||||||:|||||||||||||||||::||||**

**33F-1_cps 13617 CTCAAACAAAAAAGTTTGGGACTATGTCAATCAATTCGCTGAATTTAACAACTATATCAA 13676**

**33F_cps 13677 CTCGCCTGTAGCTAATTACAAGGGTAGCCTCTATAATCTACCTTTCAATATGAATACCTT 13736**

**:||:|||||:||:||:||:||:||:||:||:||||||||:|||||||||||||||||:||**

**33F-1_cps 13677 TTCACCTGTCGCGAACTATAAAGGAAGTCTTTATAATCTTCCTTTCAATATGAATACTTT 13736**

**33F_cps 13737 CTATGCTATGTGGGGGACAAAAACTCCTCAAGAAGTCAAAGATAAGATTACTGAGCAGAC 13796**

**|||||||||||||||||||||||||||||||||:|||||||||||||||:|||||||:||**

**33F-1_cps 13737 CTATGCTATGTGGGGGACAAAAACTCCTCAAGAGGTCAAAGATAAGATTGCTGAGCAAAC 13796**

**33F_cps 13797 AGCTGATATGAAGGATGTTGAGCCGAAAAATCTGGAAGAACAGGCTATCAAGTTGATTGG 13856**

**:|||:|:||||||||::||||:||:|||||::|||||||||||||||||||||||||:||**

**33F-1_cps 13797 GGCTCACATGAAGGACATTGAACCTAAAAACTTGGAAGAACAGGCTATCAAGTTGATCGG 13856**

**33F_cps 13857 TCCAGATGTCTATGAAAAGTTAATCAAGGGTTATACTGAAAAGCAGTGGGGACGCTCAGC 13916**

**||||||||||||||||||:|||||||||||||||||||||||||||||||||||||||||**

**33F-1_cps 13857 TCCAGATGTCTATGAAAAATTAATCAAGGGTTATACTGAAAAGCAGTGGGGACGCTCAGC 13916**

**33F_cps 13917 AACGGAACTTCCTCCATTTATCATTAAACGTCTTCCAGTTCGTTTAACATTTGATAATAA 13976**

**||||||||||||||||||||||||||||||||||||||||||||||||||||||||||||**

**33F-1_cps 13917 AACGGAACTTCCTCCATTTATCATTAAACGTCTTCCAGTTCGTTTAACATTTGATAATAA 13976**

**33F_cps 13977 CTATTTTAACGACCGTTACCAAGGAATTCCTATTGGTGGTTACAATGTCATCATCGAAAA 14036**

**||||||||||||||||||||||||||||||||||||||||||||||||||||||||||||**

**33F-1_cps 13977 CTATTTTAACGACCGTTACCAAGGAATTCCTATTGGTGGTTACAATGTCATCATCGAAAA 14036**

**33F_cps 14037 TATGCTTAAAGACGTTGAAGTTGAGCTTGGTGTTGATTTCTTTGCTCATCGTGAAGAGTT 14096**

**|||||||||||||||||||||||||||||||||||||||||||:||||||||||||||||**

**33F-1_cps 14037 TATGCTTAAAGACGTTGAAGTTGAGCTTGGTGTTGATTTCTTTACTCATCGTGAAGAGTT 14096**

**33F_cps 14097 AGAAGCATCAGCTGAAAAAGTTGTCTTTACAGGGATGATCGACCAGTATTTTGGCTACAA 14156**

**|||||||||||||||||||||||||||:|||||||||||||||||||||||||:||||||**

**33F-1_cps 14097 AGAAGCATCAGCTGAAAAAGTTGTCTTCACAGGGATGATCGACCAGTATTTTGACTACAA 14156**

**33F_cps 14157 GCACGGAGAGTTAGAATACCGTAGCCTTCGCTTTGATCATGAAATTTTGGACGAGGAAAA 14216**

**|||:||||||||||||||||||||||||||||||||||||||||||||||:|||||||||**

**33F-1_cps 14157 GCATGGAGAGTTAGAATACCGTAGCCTTCGCTTTGATCATGAAATTTTGGCCGAGGAAAA 14216**

**33F_cps 14217 TTATCAAGGAAATGCTGTAGTGAACTATACGGAGCGTGAGATTCCTTATACTCGTATTAT 14276**

**||||||||||||||||||||||||||||||||||||||||||||||||||||||||||||**

**33F-1_cps 14217 TTATCAAGGAAATGCTGTAGTGAACTATACGGAGCGTGAGATTCCTTATACTCGTATTAT 14276**

**33F_cps 14277 CGAACACAAGCATTTTGAATATGGAACACAGGCAAAGACAGTTATCACGCGTGAATATCC 14336**

**:||:||:||:||:|||||:|||||:||:||:||:||:||:||:||:||:||||||||:||**

**33F-1_cps 14277 TGAGCATAAACACTTTGAGTATGGTACGCAAGCTAAAACGGTCATTACTCGTGAATACCC 14336**

**33F_cps 14337 AGCTGACTGGAAGCGTGGGGACGAGCCTTACTATCCGATCAACGATGAGAAAAATAATGC 14396**

**||||||:|||||:||:||:||:||:|||||:|||||:|||||:|||||||:|||:|||||**

**33F-1_cps 14337 AGCTGATTGGAAACGCGGAGATGAACCTTATTATCCAATCAATGATGAGAGAAACAATGC 14396**

**33F_cps 14397 TATGTTTGCTAA---------------------GTACCAAGAGGAAGCTTCAAAGAATGA 14435**

**||||||||||||:::::::::::::::::::::|:|:::|:|:|::|:||::::|:::::**

**33F-1_cps 14397 TATGTTTGCTAAATATCAAGAAGAAGCAGCACAGAATG-ATAAGGTGATTTTCTGCGGAC 14455**

**33F_cps 14436 TAAGGTTATTTTCTGTGGACGTTTAGCAGATTATAAATATTACGATATGCATGTGGTGAT 14495**

**::::|:::::||:|::::||::::|:::|::|:|::::|||::::::::|:|::::::::**

**33F-1_cps 14456 GTTTGGCCGATTATAAATACTACGACATGCATGTGGTCATTGAACGTGCCTTAGAAGTCG 14515**

**33F_cps 14496 TGAACGGGCGCTTGAGGTTGTGGAGAAAGAGTTTGGATATGACAAAAAGTAGAATCAATT 14555**

**|:::::|::::||::|:::||:|::||::|::||:::::::|::::||::::::|::||:**

**33F-1_cps 14516 TATTAAGTGAGTTAGGAAAGTAGTCAATTACATTAAGGTCAAGTTTAATGGCCTTATATC 14575**

**33F_cps 14556 GGATAGATTTTGGAAAAGGCTTTTCCATATTTTTAGTCTTAGCAGGACATGTGTTGCTTG 14615**

**:::|::||:::|:||||::::::|::::::::::||::|||::::||:|::::|::::::**

**33F-1_cps 14576 TTTTCTATAAAGAAAAACTACAATTAGAGGAGCGAGATTTAATGTGAAAAAGATAAAAGA 14635**

**33F_cps 14616 GACTGTATCAATCGGAAAAATTTCCCACAGCAAATAACATACTATCGTTGTTGATAGCAC 14675**

**:::||::::::|:::||::||::|::::|::::|:::::::::|::::|::||:::::::**

**33F-1_cps 14636 ATATGACATTTTAAAAATTATGGCTATTATTTTAGTTGTGTTAAGTCATAGTGCGTATTA 14695**

**33F_cps 14676 AAGTCTACATATTTCATATACCAGTATTTTTTGCCTTATCAGGATACTTTTTCAAACCTG 14735**

**:|:::||:::|:::::|||::::|:||:::||::|:::::::::||::|::|||::|:::**

**33F-1_cps 14696 TAAAATATCGAGCAATTATGGTGGAATGGATTATCAACAATATTTAAATAGTCATTCAGC 14755**

**33F_cps 14736 TGTCGGATTTGAAGGAGTTCTGGCAATATGCTAAAAAGAAGACAATTGTTTTTGGTCTGC 14795**

**::|:::::|::|:|:::|:||:|::|:||::::::||:::::|:|||:||||:::::::|**

**33F-1_cps 14756 ATTTACTCTATATGAGATACTTGGTAAATTTATGGAAATTATCTATTATTTTCATATACC 14815**

**33F_cps 14796 CATATATTTTCTATTCGATCATTCACTTTGGTCTTCAAAAAGTTGCAGGGGCGACTGTTA 14855**

**::|||:|:|:::::|::::::|::|:|||::|||::::|||:::::|::|:::::||::|**

**33F-1_cps 14816 ATTATTTATGGCTATATCGGGTGTATTTTTCTCTATTCAAATAAAAAAAGATCGATGGAA 14875**

**33F_cps 14856 GAGTCCCGACGACTATATTTGATTTAATAAATATCTATAAAAATCCTATTGGAGTCTCAT 14915**

**:|:::::||::|:|:::|::::::|||:::::|:::||:||:||:::::|::::|::::|**

**33F-1_cps 14876 TAAGATTGAGAAATTATTAACTAGTAAGTTTAAAAGATTAATATTGCCATTTTTTTGTTT 14935**

**33F_cps 14916 GGTATTTATACATACTCTGGTCGATTTTGATAATGTATGGATTAGTATCTATATTTAATC 14975**

**::::|||||::::::::|:|:|::|:::::::||:|:::::|:::::::|::::||::::**

**33F-1_cps 14936 TTACTTTATTATATAGTTTGCCATTAAAATATATATCAAACTACTACAATGGTGTTTCAT 14995**

**33F_cps 14976 AAAAATCGTAGAATATTATTTTTGATAAGTGTTTTTGCTTATTGTTTAACCCTATTTGTT 15035**

**:::::::::::|::|:|::|::::::::||::||::|::::|:::::::::::::||::|**

**33F-1_cps 14996 TTTGGAGAGCTATAACTGGTCAATTCTTGTTATTAGGAAATTCTCACTTATGGTATTTGT 15055**

**33F_cps 15036 CAAACAGATATTTATATTATTCAAAGAACGCTAGTTTGGGGTATTTGTTTCTTTCTTGGA 15095**

**:::::::||:|:|::::|:|::||::|::::|::||:::::::::::::|::||||::::**

**33F-1_cps 15056 ATGCATTATTTATTATCTTTATAATTAGTTTTTATTGTTTAAGAAGAGATACTTCTATAT 15115**

**33F_cps 15096 AGTGTATTGAACGAAATTCACTTTAATAAAATTAGTTTGAAAAAATTTCTTGTTTTCTTT 15155**

**:::::::::::::::::|:::||::|:|::::::|::|::::::|||::|:::::::|::**

**33F-1_cps 15116 TTGTATACTTGTCCTTATATATTATACATGTACTGAGTTTCTTGATTCATATAACGTTAG 15175**

**33F_cps 15156 GTGATATTTGACTTTATTTATATGCTTGTATGGTTCTTATTTTATGAAGTAGAGTCTAAA 15215**

**::::|:::::|:|::|:||:::|::|||::|:::::|::::||:|:::::::|::::|||**

**33F-1_cps 15176 TAAGTGCACCATTGCAGTTTCTTTTTTGGTTTTCCATGGGATTTTTGTTCGAATCTAAAA 15235**

**33F_cps 15216 AGGGATAGTGTAAGCTATAGTAACCCAGGGGTGTGGGGGATTGCTTTTATTGTCTGTATA 15275**

**:::|::|||:|||::|:|::|:::::|:::::::::||::|:||:|||::|:::|:::::**

**33F-1_cps 15236 GAAGGAAGTATAATATTTTTTTGGAAAATCACAAATGGATTAGCCTTTTATTCTTTGTGT 15295**

**33F_cps 15276 TTAGTTGCGTTTGTAATCTTCCCTAAAATATCGGAAAAATTTCCTAAAACTTTCCTATAT 15335**

**|:::|::::|||::::::||::::|:::|:|::::||||:::::|::||::|::::|:::**

**33F-1_cps 15296 TATTTATATTTTTGGTGGTTTTAAATTTTCTATTTAAAAGCGATTTTAAAGTATTGAGTC 15355**

**33F_cps 15336 TTCACTAAATATGGTAAAGATAGTTTAGGTATCTATATCCTCCATGCACCAATTTGTAGC 15395**

**:::::|:::|::::|:|::|::::||::::::::|::|::|:::|:||::|:||:|||::**

**33F-1_cps 15356 GATTTTTTGTTGACTTATTAGCTATTTTAGGATCACTTATTTGTTACAATATTTCGTACT 15415**

**33F_cps 15396 ATGATTCGGATTCTAATGTTGAAAGTGGGAATAAACTCAGTTTTTCTTCACGTTGTTGTT 15455**

**:|:::::::||:::|:|:::::::::|::|:||||||:::|::|:::::::::::|::|:**

**33F-1_cps 15416 TTTTAAGTAATAAAACTAAAATTTTAGATAGTAAACTACTTAATCTCATTTTGATTAATG 15475**

**33F_cps 15456 GGGATTGTCTTGGGTTGGTATTTATCCATACTGGCAACTTATATATTGAAAAAAATTCCA 15515**

**::::::|::|:::::|::|:|:|:::::|:::::|:|:||:::::|::::|::::||:::**

**33F-1_cps 15476 CTTTAGGGATATATATTTTTTCTGATACTTTAAACTATTTTATATTAAGTATTTCTTATT 15535**

**33F_cps 15516 TTTTTGAATATTGTTTTATTACCACAAAAGTATATTAAATTAAAATAA------------ 15563**

**||:|:::::||:|:|||||::::||:::|:|:::|::|||||::|||:::::::::::::**

**33F-1_cps 15536 TTGTAAGTGATAGATTTATGTTTACTTCATTTGGTATAATTATTATATTTTTAATTAGAT 15595**

**33F_cps 15564 ------------------------------------------------------------ 15563**

**::::::::::::::::::::::::::::::::::::::::::::::::::::::::::::**

**33F-1_cps 15596 TTGTTTTCACTTTATTTTTGGGATTAGTCTTTACATTGCTATTTAAAAAAGTATTTCCAA 15655**

**33F_cps 15564 ----------------------- 15563**

**:::::::::::::::::::::::**

**33F-1_cps 15656 AATATTCTTGGTTAGTTAACTAG 15678**

**S4 Fig. Nucleotide alignment of 33F and 33F-1 *cps* loci.** Identical nucleotides are noted by ‘|’ and differences are highlighted by ‘:’.
