# Supplementary material for: Discovery of a Streptococcus pneumoniae serotype 33F capsular polysaccharide locus that lacks wcjE and contains a wcyO pseudogene
Source: PLoS One. 2018 Nov 5;13(11):e0206622. doi: 10.1371/journal.pone.0206622 (PMC6218050; doi:10.1371/journal.pone.0206622)
Supplement: S5 Fig — DNA sequences were aligned using MUSCLE and trees were constructed using the Tamura-Nei model in MEGA 7. Only bootstrap values above 50% are shown. (DOCX) [file pone.0206622.s007.docx]

***wzg***

***wzh***

***wzd***

***wze***

***wchA***

***wciB***

***wciD***

***wciE***

***wciF***

***wzy***


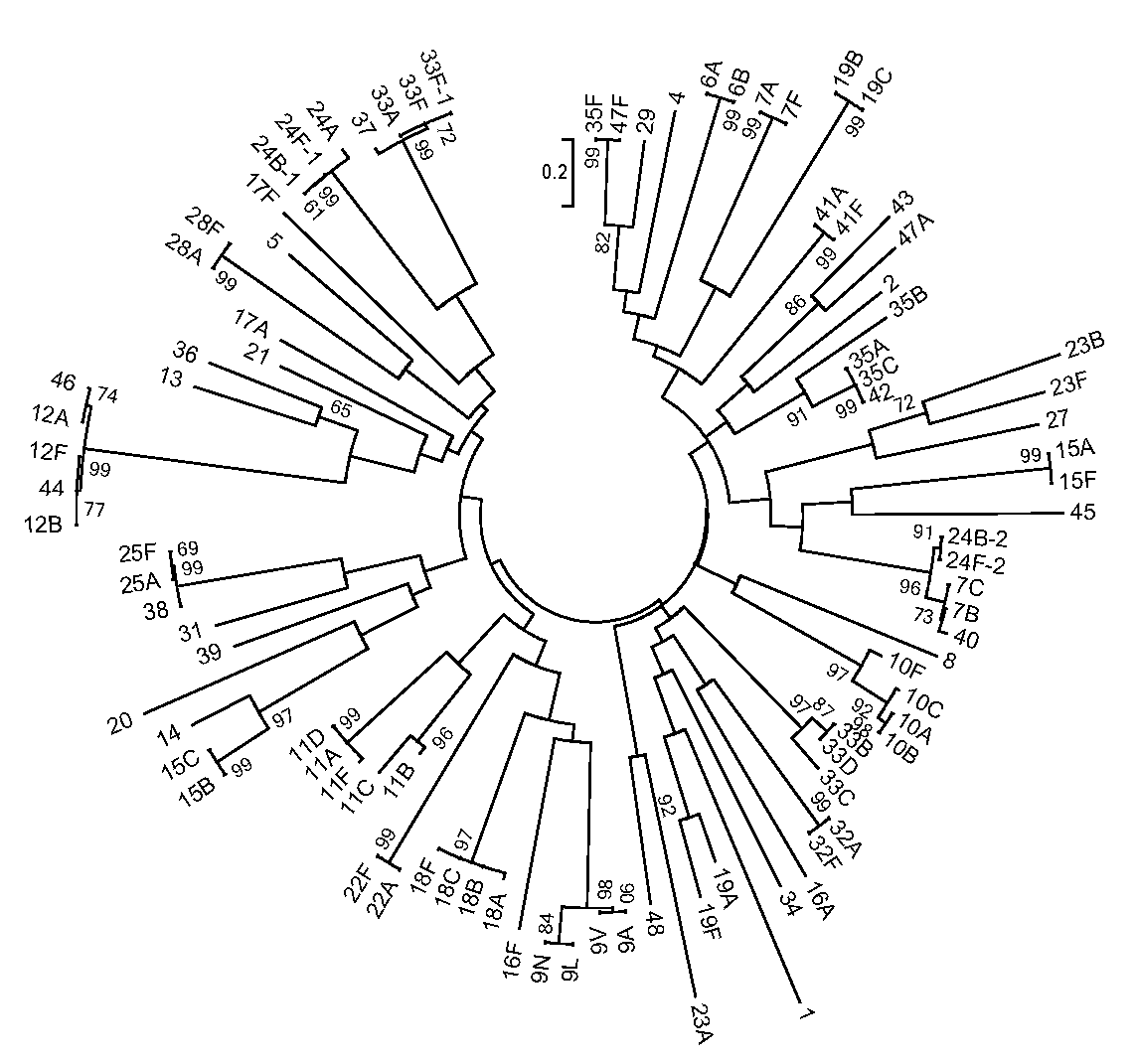


***wzx***


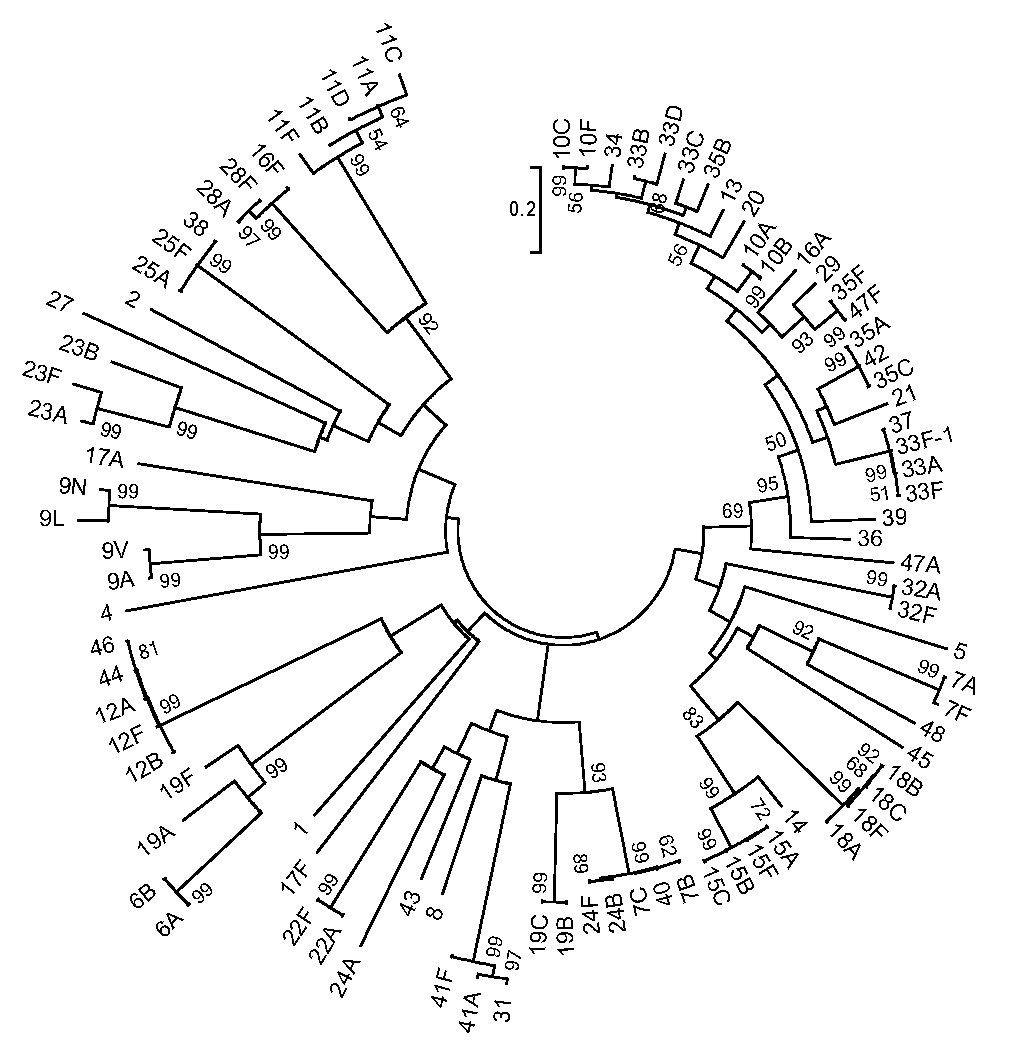


***wciG***

***glf***





***wcyO***

**S5 Fig.** **Full maximum likelihood phylogenetic trees of 33F-1 *cps* genes with homologues from all other serotypes.** DNA sequences were aligned using MUSCLE and trees were constructed using the Tamura-Nei model in MEGA 7. Only bootstrap values above 50% are shown.
